# Spatial statistical tools for genome-wide mutation cluster detection under a microarray probe sampling system

**DOI:** 10.1371/journal.pone.0204156

**Published:** 2018-09-25

**Authors:** Bin Luo, Alanna K. Edge, Cornelia Tolg, Eva A. Turley, C. B. Dean, Kathleen A. Hill, R. J. Kulperger

**Affiliations:** 1 Department of Statistical and Actuarial Sciences, Western University, London, Ontario, Canada; 2 Department of Biology, Western University, London, Ontario, Canada; 3 London Regional Cancer Program, Lawson Health Research Institute, London, Ontario, Canada; 4 Department of Statistics and Actuarial Science, University of Waterloo, Waterloo, Ontario, Canada; National Center for Biotechnology Information, UNITED STATES

## Abstract

Mutation cluster analysis is critical for understanding certain mutational mechanisms relevant to genetic disease, diversity, and evolution. Yet, whole genome sequencing for detection of mutation clusters is prohibitive with high cost for most organisms and population surveys. Single nucleotide polymorphism (SNP) genotyping arrays, like the Mouse Diversity Genotyping Array, offer an alternative low-cost, screening for mutations at hundreds of thousands of loci across the genome using experimental designs that permit capture of *de novo* mutations in any tissue. Formal statistical tools for genome-wide detection of mutation clusters under a microarray probe sampling system are yet to be established. A challenge in the development of statistical methods is that microarray detection of mutation clusters is constrained to select SNP loci captured by probes on the array. This paper develops a Monte Carlo framework for cluster testing and assesses test statistics for capturing potential deviations from spatial randomness which are motivated by, and incorporate, the array design. While null distributions of the test statistics are established under spatial randomness via the homogeneous Poisson process, power performance of the test statistics is evaluated under postulated types of Neyman-Scott clustering processes through Monte Carlo simulation. A new statistic is developed and recommended as a screening tool for mutation cluster detection. The statistic is demonstrated to be excellent in terms of its robustness and power performance, and useful for cluster analysis in settings of missing data. The test statistic can also be generalized to any one dimensional system where every site is observed, such as DNA sequencing data. The paper illustrates how the informal graphical tools for detecting clusters may be misleading. The statistic is used for finding clusters of putative SNP differences in a mixture of different mouse genetic backgrounds and clusters of *de novo* SNP differences arising between tissues with development and carcinogenesis.

## Introduction

Mutation signatures are useful tools for identifying mutagens and mutational mechanisms, and understanding genetic diversity, disease, adaptation and evolution. These signatures are identified by comparison of genomic sequences with a reference sequence and association with specific exogenous and/or endogenous conditions. Genome sequences can be viewed as a string in the genome alphabet, or equivalently as a time series or lattice sequence of a large length. For the mouse genomic experiments discussed here, the length of a single chromosome ranges from 6.14 × 10^7^ base pairs (bp) of nucleotides for chromosome 19 to 1.95 × 10^8^ bp for chromosome 1.

Current genomic technologies have broadened our perspective to mutation analysis, revealing a critically important phenomenon of non-random spacing of mutations as a new mutation signature [1]. This signature is crucial for discovery of mechanisms for mutagenesis and carcinogenesis, as well as for development of cancer treatments that target effects of driver mutations. Proximal spacing of multiple mutations has been termed ‘Kataegis’ or thundershowers of mutations [2]. Mutation showers have been reported in genomes of yeast [3, 4], mice [5, 6] and humans [7], within genes and dispersed across the genome. To date, mutation showers have been arbitrarily defined based on cancer whole genome sequencing data as the occurrence of sequence segments containing six or more consecutive mutations with an average intermutation distance of less than or equal to 1,000 bp [7]. Another definition for mutation clusters was based on empirical data for the observation of multiple mutations within 30 kb in the context of postzygotic mutations in healthy mouse tissues [6]. The largest dataset for detection of mutation showers exists for large pan-cancer studies, where mutation showers are found with low incidence in certain cancer types [7]. A chief mechanism proposed for this signature is transient hypermutagenesis, an elusive and incompletely understood phenomenon [8, 9]. Examination of the human genome for mutation showers is restricted to a very limited number of tissues or cell types and next generation sequencing. Whole genome sequencing, although the highest resolution possible, is not affordable as a population screening approach in general.

Since complete genome sequencing is expensive and generally impractical as a screening or survey method, genotyping microarrays are a low-cost alternative which are commonly used to detect mutations at loci with single nucleotide polymorphisms (SNPs). These loci are referred to as SNP sites. Differences in a single nucleotide, referred to as SNP genotype differences, can be interpreted as mutations when comparing samples. SNPs are genotyped using designed single-stranded short nucleotide probes affixed to a microarray platform. These probes complement specific locations within the genome and these locations are quite sparse in distribution across the genome relative to the genome length, yielding low cost for the array process relative to sequencing. Thus, a SNP genotype difference can be detectable or undetectable by a microarray platform, depending on whether the probes on the array are at that SNP locus. The objective we study in this paper is the development of a population, i.e., a large sample size, screening tool for a wide variety of tissues and cell types, using the low cost SNP array data for identifying clusters of putative mutations. The challenge is that arrays provide windows of observations along the genome, which depend on probe sites, in terms of both number of sites and distribution or spacing of the sites. Hence the screening tool would need to accommodate this constraint in the experimental design with microarray platforms.

**Fig 1:**
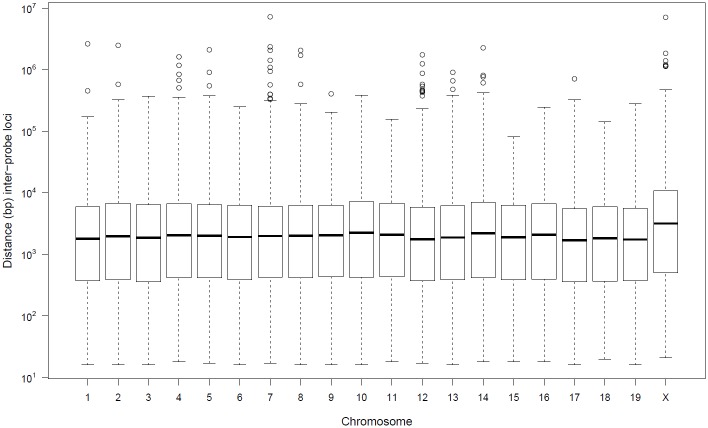
The inter-SNP locus distances (bp) for 493,290 SNP loci assayed by the probes on the Mouse Diversity Genotyping Array (MDGA) summarized for each chromosome. A boxplot of the distribution of inter-SNP locus distances (bp) for each autosome and the X chromosome.

The Mouse Diversity Genotyping Array (MDGA) is a single nucleotide polymorphism (SNP) microarray [10] that detects SNP alleles at 493,290 SNP loci [11] across the mouse genome. The alleles at each SNP locus are detected by a SNP probe set on the array. A probe set consists of eight single-stranded DNA sequences (probes) 25 bp in length. The probes are fixed to a solid surface (or chip) in a known arrangement. Due to several conditions a SNP probe needs to satisfy in design, the probes are not evenly distributed along each chromosome. To illustrate the sparsity of the probes, Fig 1 is a boxplot of the MDGA inter-SNP locus distances for each autosome and the X chromosome. The average inter-SNP locus distance is 5,210 bp, with a maximum and minimum distance of 7,268,520 bp and 16 bp, respectively. Of the SNP loci, 83.6% (412,181 SNP loci) are within 10,000 bp of another SNP locus, and 38.7% (190,714 SNP loci) are within 1,000 bp of another SNP locus. There are 22 SNP *probe deserts*, defined as consecutive probe sites spanning more than 1 million bp; the two largest gaps between consecutive probe sites are 7,268,520 bp and 7,033,330 bp on chromosomes 7 and X, respectively.

With the rapid development of genotyping and sequencing techniques in recent years, more genetic studies have begun to focus on assembling, visualizing and studying the spatial information of genomic events under different scenarios such as genome-wide association studies [12]. For cluster detection, several statistical methods have been developed and applied in DNA and protein sequencing data [3, 13–15]. Despite previous efforts for detecting clusters with sequencing data, to our knowledge, there have not been formal studies attempting to detect mutation clusters under a genotyping array system. For sequencing data, the rainfall plot has been introduced recently for visualizing the landscape of mutations [7, 16]. Specifically, a rainfall plot portrays the base pair distance of intermutation spacing along the chromosome or entire genome sequence. Here, rainfall plots are adopted to visually examine the potential existence of clusters on the whole genome or individual chromosomes for data from a mouse SNP genotyping array. Mutation clusters are suggested by low intermutation spacing values in such plots; the goal of this paper is to attach rigorous statistical inference to the identification of clusters.

From the discussion above we see that the observable microarray data depend on the probe design, that is, the locations of the probes. In this paper, we study several statistics for detecting mutation clusters: a set of non-parametric statistics based on neighbourhood measures, and a test statistic based on distances between SNP loci where mutations are detected, which is related to rainfall plots. These statistics are also studied in real-valued functional forms to summarize the cluster features. The microarray probe sampling system yields missing observations in the domain of interest. Numerical techniques have become increasingly important for the analysis of complex data structures, such as observed here. Such techniques are utilized in our analyses to incorporate the probe design constraints. The null process of complete randomness is a homogeneous Poisson process. For a natural alternative cluster process we consider the family of Neyman-Scott processes, which are a class of parent-child point processes. We evaluate the techniques through power studies which demonstrate that the tests proposed provide suitable tools for screening samples for clustering effects on the genome scale. We then apply the recommended statistical tools for finding clusters of putative SNP differences in a mixture of different mouse genetic backgrounds and for finding clusters of *de novo* SNP differences between tissues with development and with carcinogenesis.

## Methods

To detect mutation clusters genome-wide, chromosomes are studied individually as each chromosome consists of a linear space in itself. Define the set of the probe locations, determined by design, as S:S⊂R. Denote the location of the first probe target site (a SNP locus) on the chromosome as *s*_*f*_ = min_*s*∈*S*_ *s*, and the location of the last probe target site on the chromosome as *s*_*l*_ = max_*s*∈*S*_ *s*. Denote the locations of SNP genotype differences detected by the probes as *X*: *X* ⊆ *S*.

The test statistics proposed below consider SNP genotype differences within the neighborhood of a known SNP genotype difference, where neighbourhood is defined by either distance *d* from the known SNP genotype difference, or by the number of SNP differences *n*, within the neighborhood. Each statistic can be considered as a function of a specific value of *d* or *n*, or alternatively, the behavior of each statistic over a range of *d* or *n* may be considered. The summary statistics for functional behaviors utilize the well-known frameworks of the Kolmogorov-Smirnov (KS) and Cramér-von Mises (CvM) tests, adapted for this missing data context. The test statistics proposed are:

(I) Mean over all sites with SNP differences of the ratio of the number of sites with SNP genotype differences to the number of probes within fixed distance *d*
  $$\begin{array}{c} \bar{R}\left(d\right)\equiv {\bar{R}}_{S}\left(d\right)=\frac{\sum_{x\in X}\frac{N_{X}\left(x,d\right)}{N_{S}\left(x,d\right)}}{\left|X\right|} \end{array}$$ (1)
  where for arbitrary set *A*, fixed distance *d*, and site with a SNP genotype difference *x*, we have
  $$\begin{array}{c} N_{A}\left(x,d\right)=\sum_{z\in A}I\left(0<|z-x|\leq d\right) \end{array}$$ (2)
  where *I*(*E*) is the indicator function for the event *E*.
(II) Pooled mean detection ratio: the ratio of the total, over all SNP genotype differences, of the number of SNP genotype differences within distance *d* of each SNP genotype difference, to the total, over all SNP genotype differences, of the number of probes within distance *d* of each SNP genotype difference
  $$\begin{array}{c} \tilde{R}\left(d\right)\equiv {\tilde{R}}_{S}\left(d\right)=\frac{\sum_{x\in X}\;N_{X}\left(x,d\right)}{\sum_{x\in X}\;N_{S}\left(x,d\right)} \end{array}$$ (3)

The two statistics above summarize properties in the neighborhood of distance *d* from observed SNP genotype differences, while adjusting for varying probe sparsity over the chromosome. The index *S* is used to emphasize that the statistics depend on the design of the probe set *S*. While we focus on the above formulations, for comparison purposes, we also consider traditional neighbourhood formulations of test statistics:

(III) Consider *D*(*x*_0_, *n*) the minimum distance to include *n* SNP genotype differences around *x*_0_
  $$\begin{array}{c} D\left(x_{0},n\right)=\underset{d}{\inf}\{d:\sum_{x\in X}I(|x_{0}-x\left|\leq d\right)=n\} \end{array}$$ (4)
  The test statistic is the minimum of such distances over all SNP genotype differences *x*_0_ ∈ *X*,
  $$\begin{array}{c} D_{min}\left(n\right)=\min_{x_{0}\in X}D\left(x_{0},n\right) \end{array}$$ (5)
  Notice than when *n* = 2, *D*_*min*_(*n*) becomes the minimum of the distances between any two SNP genotype differences. Algorithm 1, provided at the end of this section, describes an efficient procedure for the calculation of *D*_*min*_(*n*).
(IV) Maximum of the number of SNP genotype differences within distance *d* of any given SNP genotype difference
  $$\begin{array}{c} N_{max}\left(d\right)=\max_{x\in X}N_{X}\left(x,d\right) \end{array}$$ (6)

Another test statistic proposed is a count statistic related to the distances between SNP loci with genotype differences, which are features shown in the rainfall plots. The count statistic is defined as follows:

(V) Count of inter-SNP locus distances for those SNP loci with different genotypes under threshold *d*
  $$\begin{array}{c} C\left(d\right)=\sum_{b\in B_{X}}I\left(b<d\right) \end{array}$$ (7)
  where $B_{X}={⊎}_{i=1}^{n-1}\left\{X_{\left(i+1\right)}-X_{\left(i\right)}\right\}$, and *X*_(*i*)_ is denoted as the *i*th ordered statistic in *X*, where *i* = 1, ⋯, |*X*|. The multiset *B*_*X*_ contains all of the inter-SNP locus distances for those SNP loci with different genotypes for the sample *X*.

These five statistics, generically denoted as *G*(*y*), may be viewed as function valued statistics with a fixed argument *d* or *n*. Instead of considering a fixed argument *y*, they may also be viewed as a functional form *G*(⋅), *G*(⋅) ≡ {*G*(*y*), *y* ∈ *R*(*y*)}, where *R*(*y*) is the range of *y* considered. Let G*(⋅) = *E*_0_(*G*(⋅)), the expectation of *G*(⋅) under an appropriate null hypothesis, e.g., homogeneous Poisson process, which is discussed in the next section. Two test statistics measuring the distance of *G*(⋅) from G*(⋅) as considered here are of the forms of Kolmogorov-Smirnov (KS) and Cramér-von Mises (CvM) tests [17] described as follows:

1. Kolmogorov-Smirnov test framework
  The KS test statistic is the supremum norm distance of *G* to G* over a range of *y*:
  $$\begin{array}{c} KS\left(G,G^{*}\right)=\underset{y}{\sup}\left|G\left(y\right)-G^{*}\left(y\right)\right| \end{array}$$ (8)
2. Cramér-von Mises test framework
  The CvM test statistic integrates the squared difference between *G* and G* over a range of *y*:
  $$\begin{array}{c} CvM\left(G,G^{*}\right)=\int {\left[G\left(y\right)-G^{*}\left(y\right)\right]}^{2}dy \end{array}$$ (9)

The five test statistics *G*(*y*) for specific argument *y* as described above and *KS* and *CvM* based on their functional forms *G*(⋅) are used to conduct inference.

To evaluate *KS* and *CvM*, the support of function *G*(⋅) is discretized and set as a finite grid *Y* = {*y*_*i*_, *i* = 1, ⋯, *k*}. The grid points *y*_1_ and *y*_*k*_ represent the smallest and largest values of *d* and *n* in the evaluation range respectively. Given the grid *Y*, the discrete versions of *KS* and *CvM* statistics are calculated as:

$$\begin{array}{c} \tilde{KS}\left(G,G^{*}\right)=\max_{y_{i},i=1,\cdots ,k}\left|G\left(y_{i}\right)-G^{*}\left(y_{i}\right)\right| \end{array}$$ (10)

$$\begin{array}{c} \tilde{CvM}\left(G,G^{*}\right)=\frac{1}{2}\sum_{i=1}^{k-1}\{\left\{{\left[G\left(y_{i}\right)-G^{*}\left(y_{i}\right)\right]}^{2}+{\left[G\left(y_{i+1}\right)-G^{*}\left(y_{i+1}\right)\right]}^{2}\right\}\left(y_{i+1}-y_{i}\right)\} \end{array}$$ (11)

The parameter *k* controls how dense the function *G*(⋅) is evaluated on the support [*y*_1_, *y*_*k*_]. If the selected grid points are dense, $\tilde{KS}$ and $\tilde{CvM}$ converge to *KS* and *CvM*; yet the selection of *k* should also account for feasible computational load.

**Algorithm 1: Calculation of** *D*_*min*_(*n*)

1: Let *X* = {*x*_*i*_, *i* = 1, ⋯, *K*} denote the set of ordered SNP genotype differences, where *x*_*i*_ is the *i*th ordered SNP genotype difference on the chromosome. Then there are *K* − *n* + 1 clusters of consecutive SNP genotype differences of size *n*: {{*x*_*l*_, ⋯, *x*_*l*+*n*−1_};*l* = 1, ⋯ *K* − *n* + 1}.

2: Define *D*_*l*_ ≡ min_*m*∈[*l*+1,*l*+*n*−2]_max(*x*_*m*_ − *x*_*l*_, *x*_*l*+*n*−1_ − *x*_*m*_), *l* = 1, ⋯ *K* − *n* + 1. For the *l*th cluster of SNP genotype differences, consider the set of minimum distances to include *n* *cluster SNP genotype differences* around each SNP genotype difference in the cluster; then *D*_*l*_ is the minimum distance in the set. Note that *cluster SNP genotype differences* refer to SNP genotype difference in the *l*th cluster.

3: *D*_*min*_(*n*) = min_*l*_ *D*_*l*_; *l* = 1, ⋯ *K* − *n* + 1.

## Small sample properties of the test statistics

Mutations may occur at any of the 2.8 billion base positions in the mouse genome. Among these mutations some exist at the genomic loci targeted by SNP probes and are thus detectable as SNP genotype differences by the SNP probe system, while the existence of the other mutations remains unknown. Both null and alternative hypotheses are established on underlying processes that generate all mutations, both detectable and undetectable. Since the target loci of the SNP probes are unique and non-random on each chromosome, the null and alternative distributions of the proposed test statistics are calculated conditional on the probe locations on the specific chromosome considered.

### Proposed underlying processes for the null hypothesis

Under the null hypothesis that SNP genotype differences are located at random locations along the chromosome, the underlying process generating SNP genotype differences can be assumed as a homogeneous Poisson process (hPP). Under such a process, every site on the chromosome, and in particular, every probe site, is independent and has an identical probability of having a SNP genotype difference. The relationship between the hPP rate parameter and the total expected number of detected SNP genotype differences *η* is linear. Numerical methods are adopted to obtain the null distributions of the test statistics for testing that *X*_*s*_, the observed locations of SNP genotype differences from the sample, are randomly located along the chromosome. Algorithm 2 develops the Monte Carlo estimate of the null distribution of the test statistics, while algorithm 3 provides inferential procedure.

**Algorithm 2**: **Monte Carlo estimates of the null distributions of summary statistics**

2.1: Set a finite grid *Y* = {*y*_*i*_, *i* = 1, ⋯, *k*}, which defines the scale of *d* or *n* as the evaluation range;

2.2: Simulate *M* replications of detected SNP genotype differences $\{X_{0}^{\left(m\right)},m=1,\cdots ,M\}$ from the hPP. At the *m*th replication, $X_{0}^{\left(m\right)}$ is obtained as follows:

(a): Generate the total number of underlying SNP genotype differences $N_{null}^{\left(m\right)}∼Pois\left(\hat{\lambda }\right)$, where $\hat{\lambda }$ is an estimate of the rate parameter from the observed sample *X*_*s*_: $\hat{\lambda }=\frac{(s_{l}-s_{f})\eta }{\left|S\right|}$. The parameter *η* can be set as |*X*_*s*_|, where |*A*| is the norm of set *A*, that is the count of the number of elements in *A*;
(b): Generate the set of underlying (both observable and unobservable) locations with SNP genotype differences $U_{null}^{\left(m\right)}=\left\{u_{j},j=1,\cdots ,N_{null}^{\left(m\right)}\right\}$, where independent and identically distributed random variables *u*_*j*_ ∼ *U*[*s*_*f*_, *s*_*l*_], and *U* is the discrete uniform distribution on {*s*_*f*_, ⋯, *s*_*l*_};
(c): Obtain the set of observed SNP genotype differences: $X_{0}^{\left(m\right)}=U_{null}^{\left(m\right)}\cap S$.

2.3: For each *m* = 1, ⋯ *M*, obtain $G_{X_{0}^{\left(m\right)}}\left(\cdot \right)\equiv \left\{G_{X_{0}^{\left(m\right)}}\left(y_{i}\right),i=1,\cdots ,k\right\}$ at the grid sites *y*_*i*_, *i* = 1, ⋯, *k*;

2.4: The Monte Carlo estimate of G*(⋅) is ${\hat{G}}^{*}\left(\cdot \right)\equiv \left\{\frac{1}{M}\sum_{m=1}^{M}G_{X_{0}^{\left(m\right)}}\left(y_{i}\right),i=1,\cdots ,k\right\}$;

2.5: For each *m* = 1, ⋯ *M*, calculate the $\tilde{KS}$ or $\tilde{CvM}$ test statistic:

(a): ${\tilde{KS}}_{G}^{X_{0}^{\left(m\right)}}=\tilde{KS}\left(G_{X_{0}^{\left(m\right)}},{\hat{G}}^{*}\right)$;
(b): ${\tilde{CvM}}_{G}^{X_{0}^{\left(m\right)}}=\tilde{CvM}\left(G_{X_{0}^{\left(m\right)}},{\hat{G}}^{*}\right)$;

2.6 The Monte Carlo estimates of the cumulative distribution functions of the test statistics ${\hat{F}}_{{\tilde{KS}}_{G}}$ and ${\hat{F}}_{{\tilde{CvM}}_{G}}$ are:

(a): ${\hat{F}}_{{\tilde{KS}}_{G}}\left(t\right)=\frac{1}{M}\sum_{m=1}^{M}I\left({\tilde{KS}}_{G}^{X_{0}^{\left(m\right)}}\leq t\right)$
(b): ${\hat{F}}_{{\tilde{CvM}}_{G}}\left(t\right)=\frac{1}{M}\sum_{m=1}^{M}I\left({\tilde{CvM}}_{G}^{X_{0}^{\left(m\right)}}\leq t\right)$

**Algorithm 3**: **Hypothesis testing procedure**

3.1 Based on the observed sample *X*_*s*_, calculate $G_{X_{s}}\left(\cdot \right)\equiv \left\{G_{X_{s}}\left(y_{i}\right),i=1,\cdots ,k\right\}$. The test statistics are:

(a): ${\tilde{KS}}_{G}^{X_{s}}=\tilde{KS}\left(G_{X_{s}},{\hat{G}}^{*}\right)$;
(b): ${\tilde{CvM}}_{G}^{X_{s}}=\tilde{CvM}\left(G_{X_{s}},{\hat{G}}^{*}\right)$;

3.2 Statistical inference:

(a): For hypothesis testing at significance level *α*:
  (i): KS test: if ${\tilde{KS}}_{G}^{X_{s}}>{\hat{F}}_{{\tilde{KS}}_{G}}^{-1}\left(1-\alpha \right)$, reject the null hypothesis, otherwise do not reject.
  (ii): CvM test: if ${\tilde{CvM}}_{G}^{X_{s}}>{\hat{F}}_{{\tilde{CvM}}_{G}}^{-1}\left(1-\alpha \right)$, reject the null hypothesis, otherwise do not reject.
(b): The p-values are calculated as:
  (i): KS test: $\frac{1+\sum_{m=1}^{M}I\left({\tilde{KS}}_{G}^{X_{0}^{\left(m\right)}}⩾{\tilde{KS}}_{G}^{X_{s}}\right)}{1+M}$;
  (ii): CvM test: $\frac{1+\sum_{m=1}^{M}I\left({\tilde{CvM}}_{G}^{X_{0}^{\left(m\right)}}⩾{\tilde{CvM}}_{G}^{X_{s}}\right)}{1+M}$;

The *p*-value calculation methods in step 3.2(b) of Algorithm 3 are based on the approaches for calculating *p*-values for Monte Carlo simulation provided in [18], which would yield empirical *p*-values having correct type-I error rate.

### Proposed underlying processes for alternative hypotheses

Under the alternative hypotheses, the underlying process would generate SNP genotype differences following a non-random spacing pattern. Here, the Neyman-Scott (NS) process is proposed as a suitable clustering process. The NS process is a parent-offspring process, where a cluster of several offspring is generated around each unobservable parent. The parent locations can be randomly spaced along the chromosome or follow some alternate spacing patterns. This parent-offspring type of underlying process is reasonable because it mimics a specific mutagenesis mechanism that one source of error may lead to a cluster of mutations nearby. The error source could be a binding site of a particular protein that leads to the generation of nearby mutations. This is an example of a transient state of an error-prone polymerase or a period in replication of biased dNTP pools or error-prone conditions associated with translesion bypass [5, 8, 19–22].

Three alternative hypotheses are considered, all derived from the NS parent-offspring clustering process. Each of these three alternatives differs in the domain *D*_*p*_ on which parent sites are generated as discussed below. Each parent site generates a cluster of offspring sites, with the random number of offspring following the Poisson distribution with the expected number *μ*_*o*_. The offspring sites are independent and identically distributed, truncated normal random variables centered at the parent site location. The standard deviation of the truncated normal distribution is denoted as *σ*. The half-length of the window of the truncation range is denoted as *h*.

1. Parent sites with an expected number *μ*_*p*_ are generated along the chromosome from an hPP. The domain on which parent sites are located, *D*_*p*_, is [*s*_*f*_ − *h*, *s*_*l*_ + *h*]. Only parents within this range can yield offspring detectable by the probe set, because of the truncation range in offspring distribution.
2. Parent sites are constrained to SNP probe locations: *D*_*p*_ = *S*. There are two important reasons to constrain parent sites to probe locations. First, probes are located where the corresponding SNP genotype differences have an occurrence of at least 1% in the population, so that the probe sites are selected based on their being favorable in terms of having SNP genotype differences. Secondly, under this constraint, all of the test statistics will attain the highest power compared to other parent site settings. Thus this setting is helpful for eliminating some candidate tests with sub-optimal performance.
3. The parent sites are constrained to be within a certain distance *h*_*p*_ of a probe; *D*_*p*_ = ∪_*s*∈*S*_[*s* − *h*_*p*_, *s* + *h*_*p*_]. This setting recognizes possible errors in identifying probe locations, so parents may not be exactly placed at favorable sites for SNP genotype differences.

In the simulation of each alternative hypothesis, as in the null hypothesis, the expected total number of detected SNP genotype differences *η* is set to equal the observed total SNP genotype differences |*X*_*s*_|, which is achieved by adjusting the parameters in the alternative process. Algorithm 4 details the Monte Carlo estimates of the powers.

**Algorithm 4**: **Power Study**

4.1: Set a finite grid *Y* = {*y*_*i*_, *i* = 1, ⋯, *k*} the same as in Algorithm 2;

4.2 Simulate *M*′ replications of detected SNP genotype differences $\{X_{a}^{\left(m\right)},m=1,\cdots ,M^{\prime }\}$ from a Neyman Scott process. At the *m*th replication, $X_{a}^{\left(m\right)}$ is generated as follows:

(a): Generate the total number of unobservable parent points $N_{p}^{\left(m\right)}∼Pois\left(\mu_{p}\right)$, where *μ*_*p*_ is the Poisson mean parameter.
(b): Generate the set of parent points $Z^{\left(m\right)}=\left\{z_{t}^{\left(m\right)},t=1,\cdots ,N_{p}^{\left(m\right)}\right\}$, where the iid random variable $z_{t}^{\left(m\right)}∼U\left(D_{p}\right)$ and *U* is the discrete uniform distribution on the domain *D*_*p*_.
(c): For each parent point $z_{t}^{\left(m\right)}$, generate the number of offspring $N_{ot}^{\left(m\right)}∼Pois\left(\mu_{o}\right)$, and a set of offspring $O_{t}^{\left(m\right)}=\left\{u_{tj}^{\left(m\right)},j=1,\cdots ,N_{ot}^{\left(m\right)}\right\}$, where iid random variables $u_{tj}^{\left(m\right)}∼N\left(z_{t}^{\left(m\right)},\sigma^{2}\right)$ with truncation interval $\left[z_{t}^{\left(m\right)}-h,z_{t}^{\left(m\right)}+h\right]$;
(d): Obtain the set of all generated offspring $U_{alt}^{\left(m\right)}=\cup_{t=1}^{N_{p}^{\left(m\right)}}O_{t}^{\left(m\right)}$;
(e): Obtain the set of observed SNP genotype differences $X_{a}^{\left(m\right)}:X_{a}^{\left(m\right)}=U_{alt}^{\left(m\right)}\cap S$.

4.3: For each *m* = 1, ⋯ *M*′, obtain $G_{X_{a}^{\left(m\right)}}\left(\cdot \right)\equiv \left\{G_{X_{a}^{\left(m\right)}}\left(y_{i}\right),i=1,\cdots ,k\right\}$ at the grid sites *y*_*i*_, *i* = 1, ⋯, *k*;

4.4: For each *m* = 1, ⋯ *M*′, using ${\hat{G}}^{*}\left(\cdot \right)$ from step 2.4 in Algorithm 2, calculate:

(a): ${\tilde{KS}}_{G}^{X_{a}^{\left(m\right)}}=\tilde{KS}\left(G_{X_{a}^{\left(m\right)}},{\hat{G}}^{*}\right)$;
(b): ${\tilde{CvM}}_{G}^{X_{a}^{\left(m\right)}}=\tilde{CvM}\left(G_{X_{a}^{\left(m\right)}},{\hat{G}}^{*}\right)$;

4.5 The Monte Carlo estimates of the power of the test statistics ${\hat{\beta }}_{{\tilde{KS}}_{G}}$ and ${\hat{\beta }}_{{\tilde{CvM}}_{G}}$ are as follows, where:

(a): ${\hat{\beta }}_{{\tilde{KS}}_{G}}$ = $\frac{1}{M^{\prime }}\sum_{m=1}^{M^{\prime }}I\left({\tilde{KS}}_{G}^{X_{a}^{\left(m\right)}}>{\hat{F}}_{{\tilde{KS}}_{G}}^{-1}\left(1-\alpha \right)\right)$;
(b): ${\hat{\beta }}_{{\tilde{CvM}}_{G}}$ = $\frac{1}{M^{\prime }}\sum_{m=1}^{M^{\prime }}I\left({\tilde{KS}}_{G}^{X_{a}^{\left(m\right)}}>{\hat{F}}_{{\tilde{CvM}}_{G}}^{-1}\left(1-\alpha \right)\right)$.

### Simulation parameter settings and results

Chromosome 19 is selected as an illustrative example to conduct simulation studies. Mouse 36.2 in our dataset, a mouse with a primary mammary tumor and lung metastasis, has about 50 putative *de novo* SNP genotype differences between these two tissue samples on its chromosome 19. Based on this example, the total expected number of detected SNP genotype differences *η* is chosen as 50. Under the null hypothesis, the underlying rate parameter of the hPP $\hat{\lambda }$ is calculated as 1.77 × 10^−4^ (See step 2.2(a) in Algorithm 2).

All of the statistics are evaluated using a grid of values for *d* or *n*, which are selected to be scientifically meaningful. In sequencing data, having six or more consecutive mutations with an average distance of less or equal to 1 kb is considered as a mutation shower [7]. Another definition of a mutation cluster, obtained empirically from analysis of a genic region, is having multiple mutations (2 or more) within a 30 kb region [6]. In genotyping array data, as information is missing between SNP probe sites, the evaluation range for identifying clusters would necessarily be larger than the range used in sequencing data with single base pair resolution. In this simulation study, a grid of distances *d*_*i*_, *i* = 1, ⋯, 20 are set from 5000 bp to 100, 000 bp with an interval of 5000 bp, so *d*_*i*_ = 5000*i*; while a grid of cluster sizes *n*_*i*_, *i* = 1, ⋯, 7 is set from 2 to 8 with an interval of 1, so *n*_*i*_ = *i* + 1.

Thus there are, in total, 97 statistics formulated: $\bar{R}\left(d_{i}\right),i=1,\cdots ,20$, $\tilde{R}\left(d_{i}\right),i=1,\cdots ,20$, *D*_*min*_(*n*_*i*_), *i* = 1, ⋯, 7; *N*_*max*_(*d*_*i*_), *i* = 1, ⋯, 20, *C*(*d*_*i*_), *i* = 1, ⋯, 20, and the 10 functional forms of these statistics based on *KS* or *CvM* frameworks. For each statistic, the null distribution is estimated from *M* = 10^4^ replications generated under the null process. The critical values for all tests are based on *α* = 0.05.

For the alternative processes, the parameters *σ* and *h* jointly reflect the spread of clusters of the SNP genotype differences. Here, the truncation range *h* is set as *h* = 3*σ*, as there are very low probabilities associated with the normal distribution outside this range. In the definition of *D*_*p*_ in alternative hypothesis (3), *h*_*p*_ is set as *h*_*p*_ = *σ*; note that *h*_*p*_ = +∞ for alternative hypothesis (1), where *h*_*p*_ = 0 for alternative hypothesis (2). The simulation study evaluates power performance of all test statistics with two factors, *μ*_*o*_ and *σ*. With *μ*_*o*_ and *σ* specified, the parameter *μ*_*p*_ is set to ensure that the expected number of detected SNP genotype differences *η* = 50. The experiment adopts a full factorial design with: (i) *μ*_*o*_ having two levels, 375 and 1125, denoting low and high levels of offspring within a cluster in order that powers of the statistics being evaluated are away from the extremes of 0 and 1, so that the performance of the test statistics can be differentiated; and (ii) *σ* having levels of grid distances of 500 bp, and from 1000 bp to 10000 bp with increment of 1000 bp. These values of *σ* are based on the definition of a mutation cluster by [6]; i.e., the truncation range 6*σ* ranges from 3kb to 60kb.

**Fig 2:**
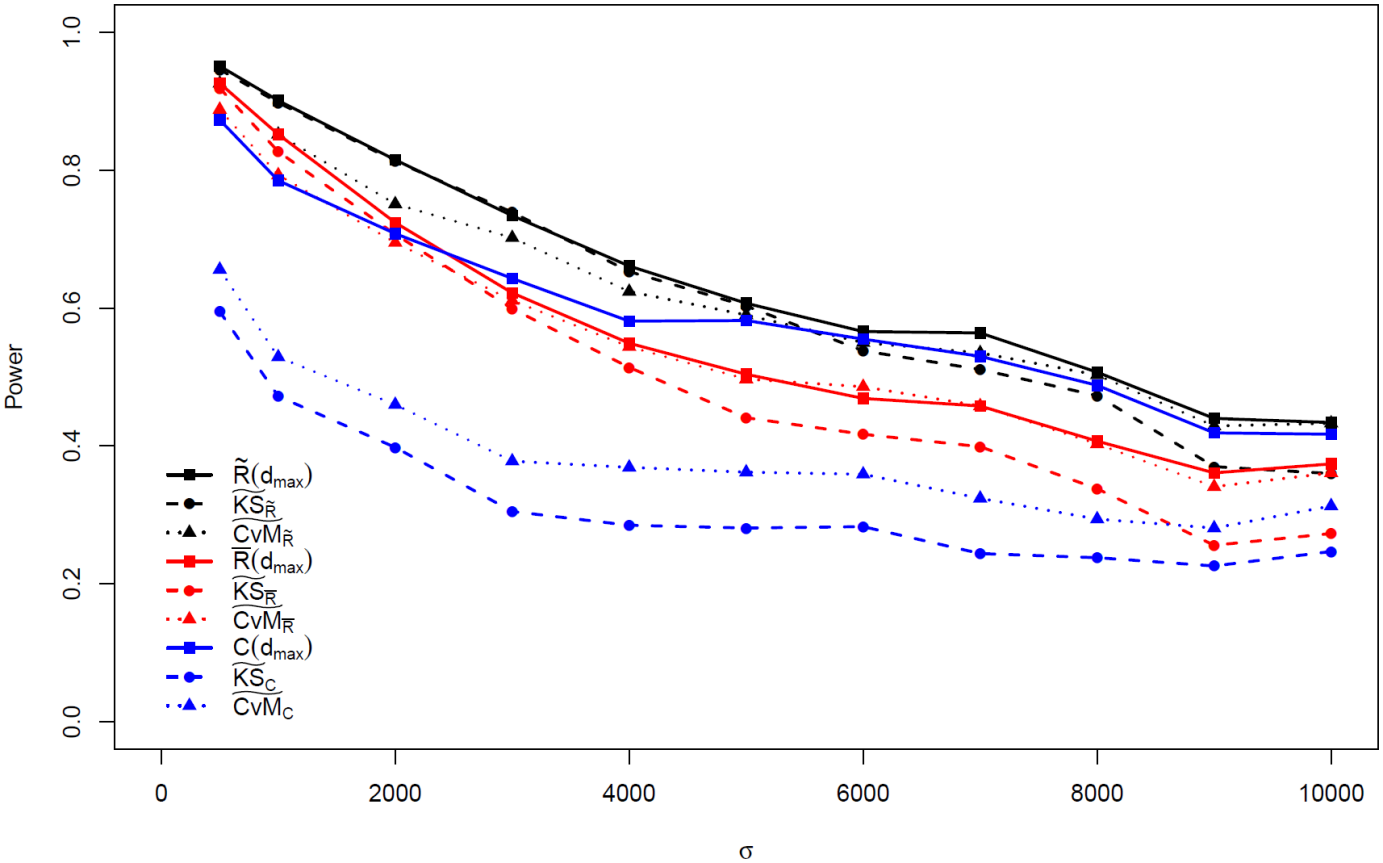
Power performance of statistics related to $\bar{R}\left(d\right)$, $\tilde{R}\left(d\right)$, and *C*(*d*) under alternative hypothesis (1) with parameter *μ*_*o*_ = 375. Only maximum powers of $\bar{R}\left(d\right)$, $\tilde{R}\left(d\right)$, and *C*(*d*) over values of *d* considered are displayed; *d*_*max*_ refers to the value of *d* yielding the largest power.

In Supplementary Information, S1–S3 Tables provide power results for *μ*_*o*_ = 375 under each of the three alternative hypotheses, while S4–S6 Tables provide associated results for *μ*_*o*_ = 1125. In each table, for the test statistics with fixed argument of *d* or *n*, only the highest powers across all the arguments are displayed. These tables in the Supplementary Information provide the identical information as in Fig 2, and S2–S5 Figs, and also provide additional details on the lower performance of two statistics that are not displayed. The optimal argument settings for all five statistics with fixed arguments to achieve highest powers across various parameter settings of *σ* are available in S7–S9 Tables for *μ*_*o*_ = 375, and S10–S12 Tables for *μ*_*o*_ = 1125, under each of the three alternative hypotheses respectively.

Under the alternative hypothesis (1), for *μ*_*o*_ = 375, in general, the power of each test statistic decreases as *σ* increases. The test statistics based on $\tilde{R}\left(d\right)$, $\bar{R}\left(d\right)$ and *C*(*d*) generally have higher powers than those based on *N*_*max*_(*d*) and *D*_*min*_(*n*). Fig 2 contrasts the power performance of nine categories of statistics based on $\tilde{R}\left(d\right)$, $\bar{R}\left(d\right)$ and *C*(*d*), including the statistics with fixed arguments as well as their function forms. Among these nine, $\tilde{R}\left(d\right)$ has the highest power and outperforms $\bar{R}\left(d\right)$ and *C*(*d*) in all the settings of *σ*. Among the six functional forms of statistics, ${\tilde{CvM}}_{\tilde{R}}$ and ${\tilde{KS}}_{\tilde{R}}$ outperform the other four functional forms of statistics, and ${\tilde{CvM}}_{\tilde{R}}$ has better power performance than ${\tilde{KS}}_{\tilde{R}}$ as *σ* increases.

The power performance under alternatives (2) and (3) for *μ*_*o*_ = 375, available in S1 and S2 Figs, provide similar results to that described for alternative hypothesis (1). Power is generally highest under alternative (2) and lowest under alternative (1) given all the other settings remain constant. One noticeable difference from alternative hypotheses (2) and (3) compared to (1) is that the powers of *C*(*d*) outperform $\tilde{R}\left(d\right)$ when *σ* is not small. The comparison among the six functional forms of statistics shows similar results for alternative hypothesis (1).

For *μ*_*o*_ = 1125, the powers of the test statistics are higher than when *μ*_*o*_ = 375. The powers are closer to 1 and decrease less dramatically over *σ* than for the cases where *μ*_*o*_ = 375. The patterns of power comparisons are similar to the cases where *μ*_*o*_ = 375. Yet the powers of $\tilde{R}\left(d\right)$ are comparable with *C*(*d*) when *σ* is large and both are quite close to 1 under alternative hypotheses (2) and (3). The power performance of the statistics under the three alternative hypotheses for *μ*_*o*_ = 1125 is available in S3–S5 Figs.

**Fig 3:**
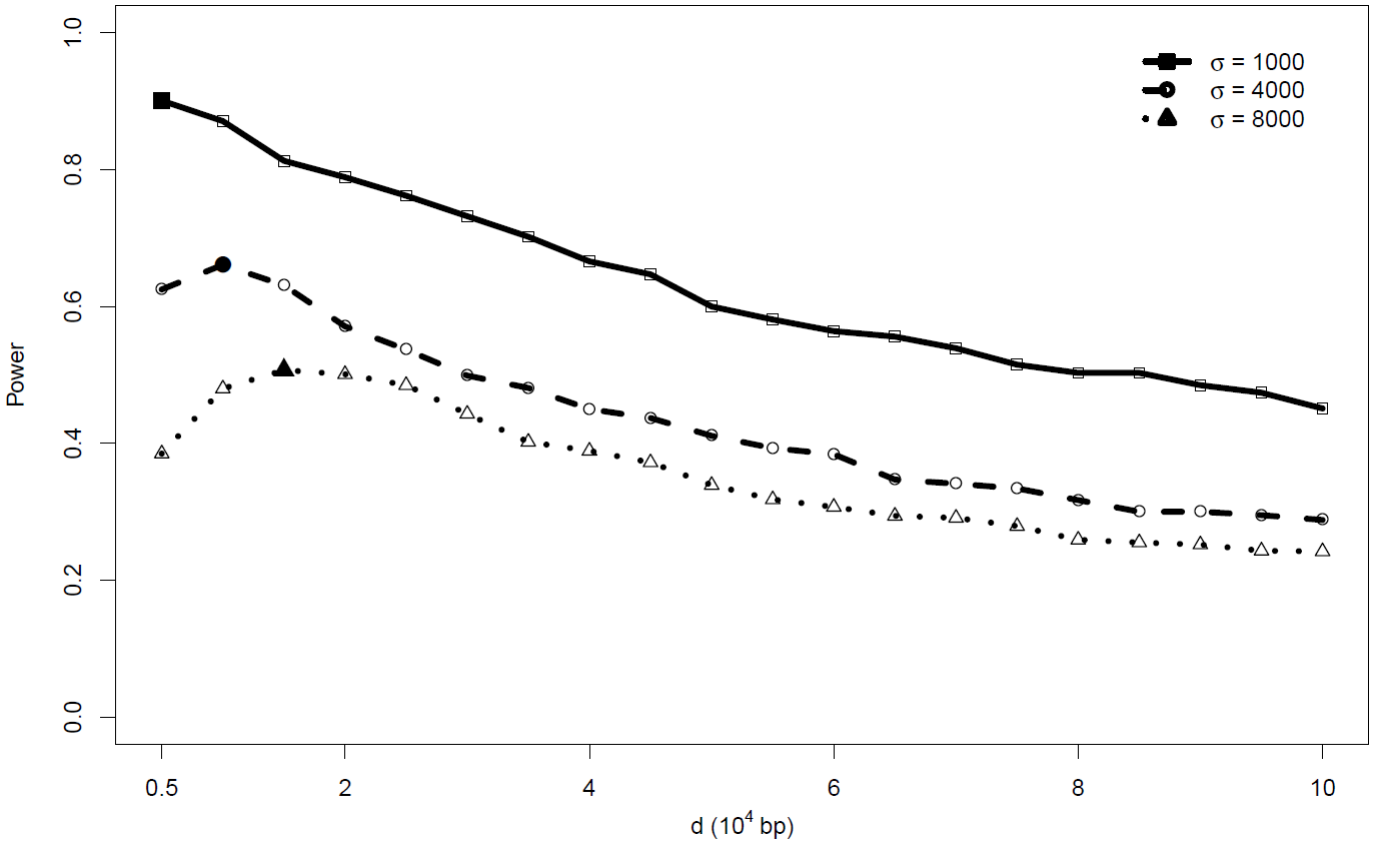
Power performance of test statistics $\tilde{R}\left(d\right)$ across a grid of *d* under alternative hypothesis (1) with parameter *μ*_*o*_ = 375. The solid points indicate the maximum power for the particular parameter setting.

The power performance of $\tilde{R}\left(d\right)$ and *C*(*d*) seem to be best among the nine categories of statistics, yet they suffer the disadvantage that they require a choice of *d*. The optimal argument choices of *d* are usually unknown in application. Moreover, the optimal choices of *d* may change over parameter settings, particularly for *σ*, as seen in Fig 3 for $\tilde{R}\left(d\right)$. Importantly, using a sub-optimal choice of *d* can yield very low power.

In conclusion, the functional statistic ${\tilde{CvM}}_{\tilde{R}}$ is the preferred test statistic in applications because of its general high power performance, oftentimes close to the best among all statistics; importantly, with this statistic no specific choice of tuning parameter *d* needs to be defined.

## Application

### Genotyping method

DNA was extracted from mouse tissue samples using the Wizard^®^ Genomic DNA Purification Kit (Promega, Madison, WI). Isolated DNA was submitted to the London Regional Genomics Centre to be processed (restriction enzyme digested, amplified, fragmented and fluorescently labeled) and hybridized to the Mouse Diversity Genotyping Array (MDGA; Affymetrix^®^, Santa Clara, CA) [10]. Genotyping was performed for each of the three specific examples within the context of separate experimental designs with a minimum cohort size of 12 samples and a maximum of 351 samples. Genotyping Console (Affymetrix^®^, Santa Clara, CA) was used to call genotypes at the 493,290 SNP loci represented by the MDGA, using the fluorescence intensity data. The Genotyping Console software uses a clustering algorithm, Birdseed v2, and assigns each SNP locus as 1 of 4 possible calls: AA (homozygous for the most common allele), AB (heterozygous, one of each allele), BB (homozygous for the less common allele), or no call if the SNP genotype calls did not cluster well with any of the three possible genotypes. The resulting data for each biological sample used for further analysis consist of a list of SNP genotype calls, their locations in the genome (chromosome number and base pair number) and the genotyping call given by Genotyping Console for each sample. In the data sets utilized for testing for existence of clusters in this paper, the events are defined as SNP genotype differences, which are the binary indicators of differences at SNP loci when contrasting two biological samples. The genotyping call and the consequent SNP genotype differences are putative until the genotyping is confirmed by an alternate technology. All animal work was conducted according to relevant national and international guidelines. Western University’s Animal Use Subcommittee approved the study. All guidelines were followed including those approved standard operating procedures for euthanasia.

### Analyses for three biological samples of interest

Three specific examples are considered here.

1. Detection of known clusters of putative SNP genotype differences in a mouse with a known mixed genetic background;
2. Test for the existence of clusters of putative SNP genotype differences arising postzygotically between two healthy tissues from a C57BL/6J mouse;
3. Test for the existence of clusters in comparison of two cancerous tissues from a MMTV-PyMT transgenic mouse [23].

**Fig 4:**
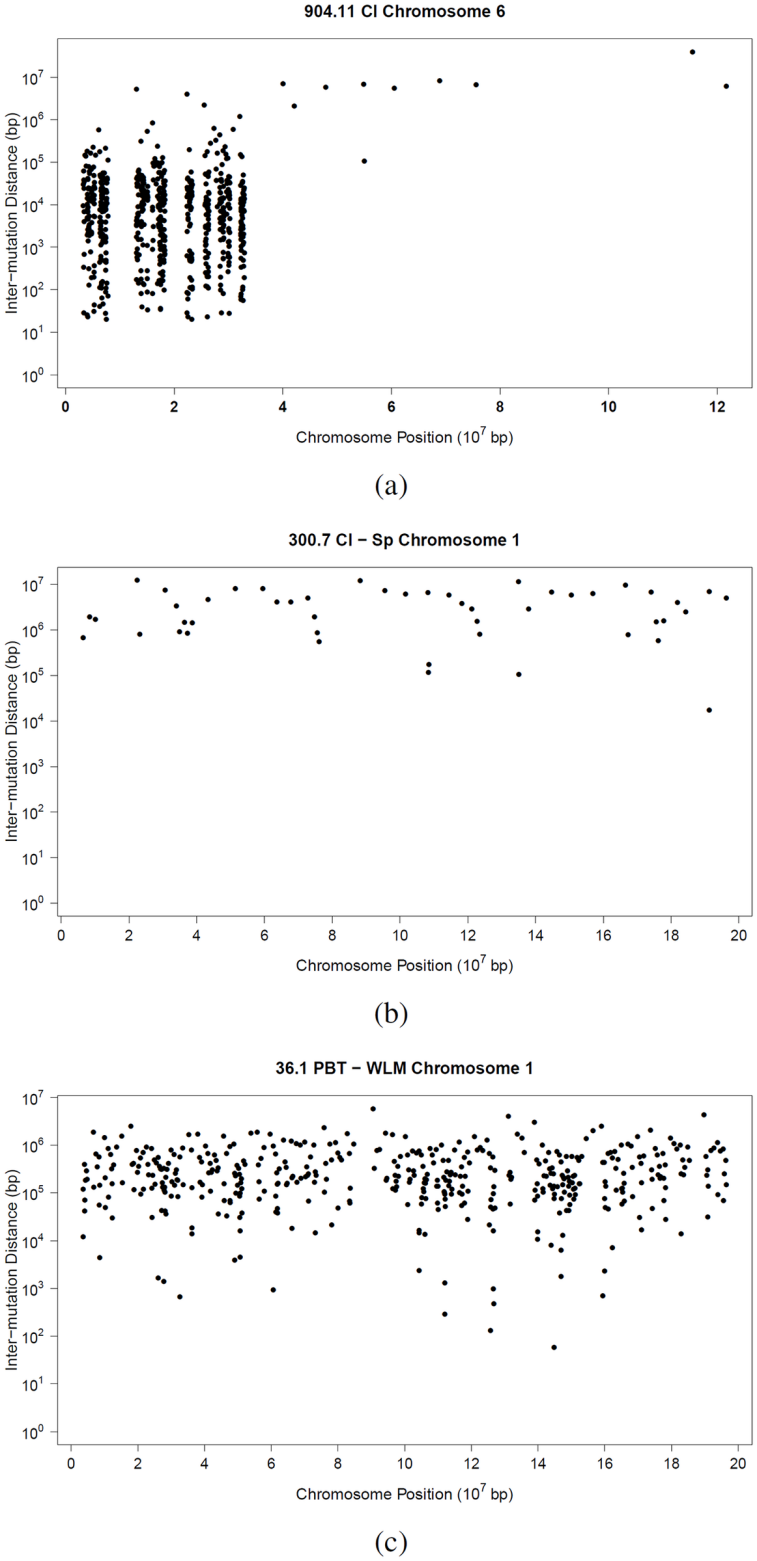
Rainfall plots portraying the SNP genotype differences due to mixed genetic background, putative new mutations arising during development of two normal tissues of the same mouse and putative mutations arising between two cancerous tissues from the same mouse. (A) Rainfall plot for chromosome 6 from a mouse (identifier: 904.11) with mixed genetic background (75% C57BL/6J and 25% CBA/CaJ). (B) Rainfall plot for chromosome 1 for a comparison of normal cerebellum and spleen tissue from the same mouse (identifier: 300.7). (C) Rainfall plot for chromosome 1 for comparison of primary mammary tumor and lung tissue with metastases from a MMTV-PyMT transgenic mouse (mouse identifier 36.1). (Legend: Cl cerebellum, Sp spleen, PMT primary mammary tumor, WLM whole lung with metastases).

Rainfall plots portraying the mutation landscapes of the three samples are provided in Fig 4. On a rainfall plot, each point represents a single mutation with its distance (in base pairs) to the previous mutation in log scale plotted on the y axis, and the base pair location in the genome is plotted on the x axis. Rainfall plots display mutations detected along a single chromosome or potentially across the entire genome. Although the plots offer a helpful visualization of the data, they do not provide formal evidence of clustering [16].

As an example of a positive control for known clustered putative SNP genotype differences in a genome, the recommended ${\tilde{CvM}}_{\tilde{R}}$ test statistic was used to analyze SNP genotype differences in normal cerebellar tissue from a mouse with a known mixed genetic background of two common inbred mouse strains (75% C57BL/6J and 25% CBA/CaJ), example 1. For chromosome 6 (Fig 4A), the test statistic rejects the null hypothesis at a significance level of 0.05, indicating existence of mutation clusters along the chromosome.

In example 2, the ${\tilde{CvM}}_{\tilde{R}}$ test statistic was used to analyze SNP genotype differences along chromosome 1 between cerebellar and splenic tissue from a healthy C57BL/6J inbred mouse (Fig 4B). The SNP genotype differences detected are hypothesized to have arisen by spontaneous mutation mechanisms resulting in somatic mutations propagated with cell division during development. The test statistic failed to reject the null hypothesis at the significance level of 0.05, indicating no existence of clusters of putative SNP genotype differences along the chromosome.

In the third example, the ${\tilde{CvM}}_{\tilde{R}}$ test statistic was used to analyze SNP genotype differences observed along chromosome 1 for a comparison of primary mammary tumor and lung tissue with metastases from the same MMTV-PyMT transgenic mouse (Fig 4C). The test statistic rejects the null hypothesis at a significance level of 0.05, indicating existence of mutation clusters along the chromosome. As mentioned in [16], the interpretation of rainfall plots is difficult and subject to pitfalls. The example in Fig 4C shows that when a subjective judgment from a visual examination of the rainfall plot is ambiguous and inconclusive, the rigorous statistical tool developed here can provide an objective decision-making approach for detecting the existence of mutation clusters.

## Discussion

In order to perform rigorous statistical testing to detect existence of clusters of putative SNP genotype differences identified by genotyping array probe systems, 97 candidate test statistics are proposed and evaluated. Conditional null distributions of test statistics are obtained by Monte Carlo simulations. The powers of all the test statistics are studied under three different types of Neyman-Scott processes, intended to mimic the unknown underlying mutation generation mechanisms. Various choices of parameters for alternative hypotheses are used to evaluate the power performance of the candidate statistics. Among all of the parameter settings, the Cramér-von Mises version of the pooled ratio estimate (${\tilde{CvM}}_{\tilde{R}}$) has high power among all candidate tests and lacks dependence on optimal argument choices. It also possesses the desirable property of having power performance degrade less over various parameter settings as the cluster range becomes larger. The functional form of the *C*(*d*) statistic based on the rainfall plot performs substantially poorer. Therefore ${\tilde{CvM}}_{\tilde{R}}$ is recommended as an effective statistic for detection of clustering.

The test statistics are developed conditional on the probe design and total number of detected SNP genotype differences. When applied to a new scenario, the null distributions of all the statistics need to be established according to the specific probe design on a chromosome and total number of detected SNP genotype differences using Algorithm 2. The rate parameter of hPP under the null hypothesis can be estimated from a single chromosome of interest without the need of extra information from other chromosomes in the same biological sample or any other replicates. However, it can also be estimated from several chromosomes under a justified experimental setting. For example, the rate parameter can be estimated from certain replicates which can be assumed to share a common underlying mutation rate under certain experimental conditions. When the objective is to carry out the mutation cluster detection genome-wide, all of the chromosomes in a sample should be tested separately. Multiple testing issues arise when the statistic is applied to multiple chromosomes from either one or a number of biological samples. These multiple tests can be independent or correlated depending on the biological context. In order to achieve a desirable overall type I error rate or false discovery rate (FDR), statistical methods such as the Bonferroni correction or by [24] may be applied to achieve desirable testing properties, depending on the goal of the research.

The methods developed in this article are designed for cluster detection under a genotyping array probe design. The probe design provides a cost-effective way for mutation detection compared to sequencing every base pair of the entire genome. Instead of a high resolution of mapping of mutations in the genome, the probe system usually only reveals a small proportion of information on a chromosome, leaving the regions outside of the probe sites unknown. As mutations in regions where probes are absent are undetectable by design, any mutation clusters occurring in such regions are correspondingly undetectable. The test statistics are established based on the information on the probe system, so they can only identify clustering when the probe system is capable of detecting potential clusters. The power evaluations in this study are conditional on the existence of the underlying clusters generated from a known clustering mechanism. This mechanism does not necessarily guarantee that clusters are detectable by the specific probe system. If all the samples evaluated in the power studies contained clusters detectable by the probe systems, the power performances of the tests would most likely be higher. One of the reasons for some low power performances in certain alternative parameter settings may be that clusters generated are not detected by the probe system. Designing an array with a larger number of probes or switching to an existing array system with a larger number of probes will augment the probability of detecting existing clusters.

In studies involving known genetic backgrounds, prior information on detected SNP differences may be utilized to improve the power of testing for mutation clusters. For example, information on SNP differences in high linkage disequilibrium (LD) with more unobserved SNP differences in their neighborhood may be given greater weight in the testing procedure. Alternatively, information on SNP genotypes undetectable by the microarray platform may be inputed based on other information such as known haplotypes [25]. However, for studies with *de novo* mutations, such as in healthy somatic tissues and in cancer studies, the imputation based on LD or known haplotypes may not be appropriate; even so, other prior knowledge may become helpful. Extensions of the methods discussed in this paper could incorporate improvements based on such prior knowledge.

After mutation clusters have been detected, different downstream analyses are possible. The nature of the mutation types in clusters can be used to identify mutation signatures and to infer the underlying mutational mechanisms. Alternatively, the mutation clusters can be linked to functional annotations for the genome and inferences can be made about the functional impact of the mutation clusters.

The method can be generalized to any one dimensional system where every site is observed, such as DNA or protein sequencing data, with probes designated as having length one at each site of the system. The method can be applied to cluster detection of any single site event along any one dimensional system, as for example, the distribution of DNA methylation locations detected by the CpG site probe system as described by [12].

The arbitrary and informal graphical tools and definitions for portraying and detection mutation clusters can now be replaced with a formal statistic test for mutation cluster detection. The recommended test statistics in this study provide tools for genome-wide detection of mutation clusters under the genotyping probe system. Due to the cost-effectiveness of array systems, larger scales of experimental designs can be adopted compared to those possible with next generation sequencing techniques. Certain samples with putative mutation clusters can be further confirmed and investigated by sequencing techniques.

## Supporting information

S1 FigPower performance of statistics related to $\bar{R}\left(d\right)$, $\tilde{R}\left(d\right)$, and *C*(*d*) under alternative hypothesis (2) with parameter *μ*_*o*_ = 375.Only maximum powers of $\bar{R}\left(d\right)$, $\tilde{R}\left(d\right)$, and *C*(*d*) over values of *d* considered are displayed; *d*_*max*_ refers to the value of *d* yielding the largest power.(TIF)

S2 FigPower performance of statistics related to $\bar{R}\left(d\right)$, $\tilde{R}\left(d\right)$, and *C*(*d*) under alternative hypothesis (3) with parameter *μ*_*o*_ = 375.Only maximum powers of $\bar{R}\left(d\right)$, $\tilde{R}\left(d\right)$, and *C*(*d*) over values of *d* considered are displayed; *d*_*max*_ refers to the value of *d* yielding the largest power.(TIF)

S3 FigPower performance of statistics related to $\bar{R}\left(d\right)$, $\tilde{R}\left(d\right)$, and *C*(*d*) under alternative hypothesis (1) with parameter *μ*_*o*_ = 1125.Only maximum powers of $\bar{R}\left(d\right)$, $\tilde{R}\left(d\right)$, and *C*(*d*) over values of *d* considered are displayed; *d*_*max*_ refers to the value of *d* yielding the largest power. *σ*.(TIF)

S4 FigPower performance of statistics related to $\bar{R}\left(d\right)$, $\tilde{R}\left(d\right)$, and *C*(*d*) under alternative hypothesis (2) with parameter *μ*_*o*_ = 1125.Only maximum powers of $\bar{R}\left(d\right)$, $\tilde{R}\left(d\right)$, and *C*(*d*) over values of *d* considered are displayed; *d*_*max*_ refers to the value of *d* yielding the largest power. *σ*.(TIF)

S5 FigPower performance of statistics related to $\bar{R}\left(d\right)$, $\tilde{R}\left(d\right)$, and *C*(*d*) under alternative hypothesis (3) with parameter *μ*_*o*_ = 1125.Only maximum powers of $\bar{R}\left(d\right)$, $\tilde{R}\left(d\right)$, and *C*(*d*) over values of *d* considered are displayed; *d*_*max*_ refers to the value of *d* yielding the largest power. *σ*.(TIF)

S1 TablePower of the tests under alternative hypothesis (1) with *μ*_*o*_ = 375 under various *σ* choices.Under each parameter setting, *h* is set as *h* = 3*σ* and *μ*_*p*_ is set to match with *η* = 50. For $\bar{R}\left(d\right)$, $\tilde{R}\left(d\right)$, *D*_*min*_(*n*), *N*_*max*_(*d*) and *C*(*d*), only the maximum power across the values considered for *d* or *n* is shown. The significance level of the test is set as *α* = 0.05.(PDF)

S2 TablePower of the tests under alternative hypothesis (2) with *μ*_*o*_ = 375 under various *σ* choices.Under each parameter setting, *h* is set as *h* = 3*σ* and *μ*_*p*_ is set to match with *η* = 50. For $\bar{R}\left(d\right)$, $\tilde{R}\left(d\right)$, *D*_*min*_(*n*), *N*_*max*_(*d*) and *C*(*d*), only the maximum power across the values considered for *d* or *n* is shown. The significance level of the test is set as *α* = 0.05.(PDF)

S3 TablePower of the tests under alternative hypothesis (3) with *μ*_*o*_ = 375 under various *σ* choices.Under each parameter setting, *h* is set as *h* = 3*σ* and *μ*_*p*_ is set to match with *η* = 50. For $\bar{R}\left(d\right)$, $\tilde{R}\left(d\right)$, *D*_*min*_(*n*), *N*_*max*_(*d*) and *C*(*d*), only the maximum power across the values considered for *d* or *n* is shown. The significance level of the test is set as *α* = 0.05.(PDF)

S4 TablePower of the tests under alternative hypothesis (1) with *μ*_*o*_ = 1125 under various *σ* choices.Under each parameter setting, *h* is set as *h* = 3*σ* and *μ*_*p*_ is set to match with *η* = 50. For $\bar{R}\left(d\right)$, $\tilde{R}\left(d\right)$, *D*_*min*_(*n*), *N*_*max*_(*d*) and *C*(*d*), only the maximum power across the values considered for *d* or *n* is shown. The significance level of the test is set as *α* = 0.05.(PDF)

S5 TablePower of the tests under alternative hypothesis (2) with *μ*_*o*_ = 1125 under various *σ* choices.Under each parameter setting, *h* is set as *h* = 3*σ* and *μ*_*p*_ is set to match with *η* = 50. For $\bar{R}\left(d\right)$, $\tilde{R}\left(d\right)$, *D*_*min*_(*n*), *N*_*max*_(*d*) and *C*(*d*), only the maximum power across the values considered for *d* or *n* is shown. The significance level of the test is set as *α* = 0.05.(PDF)

S6 TablePower of the tests under alternative hypothesis (3) with *μ*_*o*_ = 1125 under various *σ* choices.Under each parameter setting, *h* is set as *h* = 3*σ* and *μ*_*p*_ is set to match with *η* = 50. For $\bar{R}\left(d\right)$, $\tilde{R}\left(d\right)$, *D*_*min*_(*n*), *N*_*max*_(*d*) and *C*(*d*), only the maximum power across the values considered for *d* or *n* is shown. The significance level of the test is set as *α* = 0.05.(PDF)

S7 Table Optimal argument settings under alternative hypothesis (1) with *μ*_*o*_ = 375. Optimal argument settings of *d* or *n* for Neyman-Scott (NS) process under alternative hypothesis (1) with *μ*_*o*_ = 375 under various *σ* choices. Under each parameter setting, *h* is set as *h* = 3*σ* and *μ*_*p*_ is set to match with *η* = 50.
(PDF)

S8 Table Optimal argument settings under alternative hypothesis (2) with *μ*_*o*_ = 375. Optimal argument settings of *d* or *n* for Neyman-Scott (NS) process under alternative hypothesis (2) with *μ*_*o*_ = 375 under various *σ* choices. Under each parameter setting, *h* is set as *h* = 3*σ* and *μ*_*p*_ is set to match with *η* = 50.
(PDF)

S9 Table Optimal argument settings under alternative hypothesis (3) with *μ*_*o*_ = 375. Optimal argument settings of *d* or *n* for Neyman-Scott (NS) process under alternative hypothesis (3) with *μ*_*o*_ = 375 under various *σ* choices. Under each parameter setting, *h* is set as *h* = 3*σ* and *μ*_*p*_ is set to match with *η* = 50.
(PDF)

S10 Table Optimal argument settings under alternative hypothesis (1) with *μ*_*o*_ = 1125. Optimal argument settings of *d* or *n* for Neyman-Scott (NS) process under alternative hypothesis (1) with *μ*_*o*_ = 1125 under various *σ* choices. Under each parameter setting, *h* is set as *h* = 3*σ* and *μ*_*p*_ is set to match with *η* = 50.
(PDF)

S11 Table Optimal argument settings under alternative hypothesis (2) with *μ*_*o*_ = 1125. Optimal argument settings of *d* or *n* for Neyman-Scott (NS) process under alternative hypothesis (2) with *μ*_*o*_ = 1125 under various *σ* choices. Under each parameter setting, *h* is set as *h* = 3*σ* and *μ*_*p*_ is set to match with *η* = 50.
(PDF)

S12 Table Optimal argument settings under alternative hypothesis (3) with *μ*_*o*_ = 1125. Optimal argument settings of *d* or *n* for Neyman-Scott (NS) process under alternative hypothesis (3) with *μ*_*o*_ = 1125 under various *σ* choices. Under each parameter setting, *h* is set as *h* = 3*σ* and *μ*_*p*_ is set to match with *η* = 50.
(PDF)

S1 Dataset SNP loci positions over the entire mouse genome on MDGA. (CSV)

S2 Dataset Data example 1. Chromosomal positions of SNP genotype differences in normal cerebellar tissue from chromosome 6 of a mouse with a known mixed genetic background of two common inbred mouse strains (75% C57BL/6J and 25% CBA/CaJ).
(CSV)

S3 Dataset Data example 2. Chromosomal positions of SNP genotype differences along chromosome 1 between cerebellar and splenic tissue from a healthy C57BL/6J inbred mouse.
(CSV)

S4 Dataset Data example 3. Chromosomal positions of SNP genotype differences observed along chromosome 1 for a comparison of primary mammary tumor and lung tissue with metastases from the same MMTV-PyMT transgenic mouse.
(CSV)
