# Supplementary figures and images for: Spatial statistical tools for genome-wide mutation cluster detection under a microarray probe sampling system
Source: PLoS One. 2018 Sep 25;13(9):e0204156. doi: 10.1371/journal.pone.0204156 (PMC6155535; doi:10.1371/journal.pone.0204156)

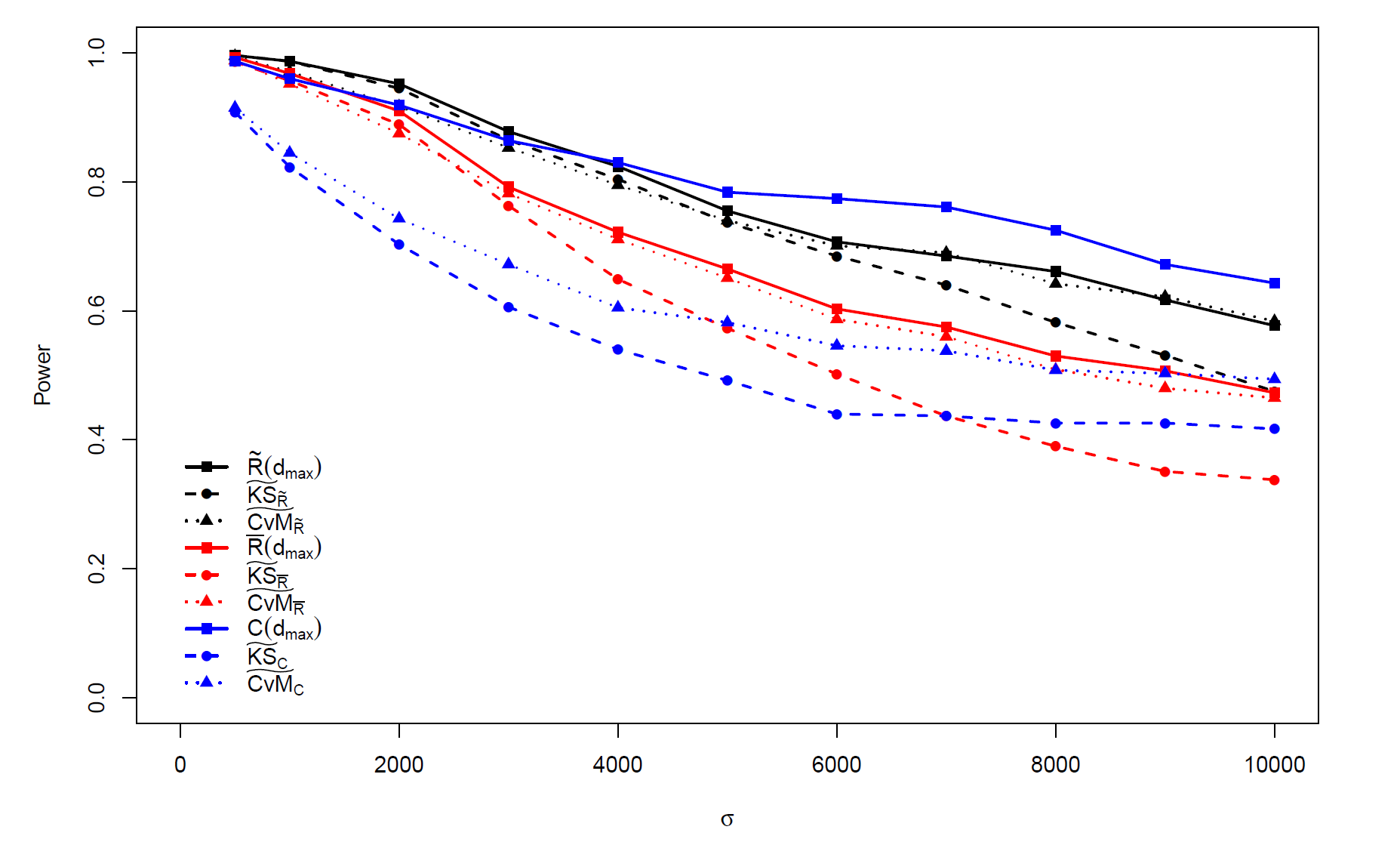

Supplement: S1 Fig — Only maximum powers of R¯(d), R˜(d), and C(d) over values of d considered are displayed; dmax refers to the value of d yielding the largest power. (TIF) [file pone.0204156.s001.TIF]

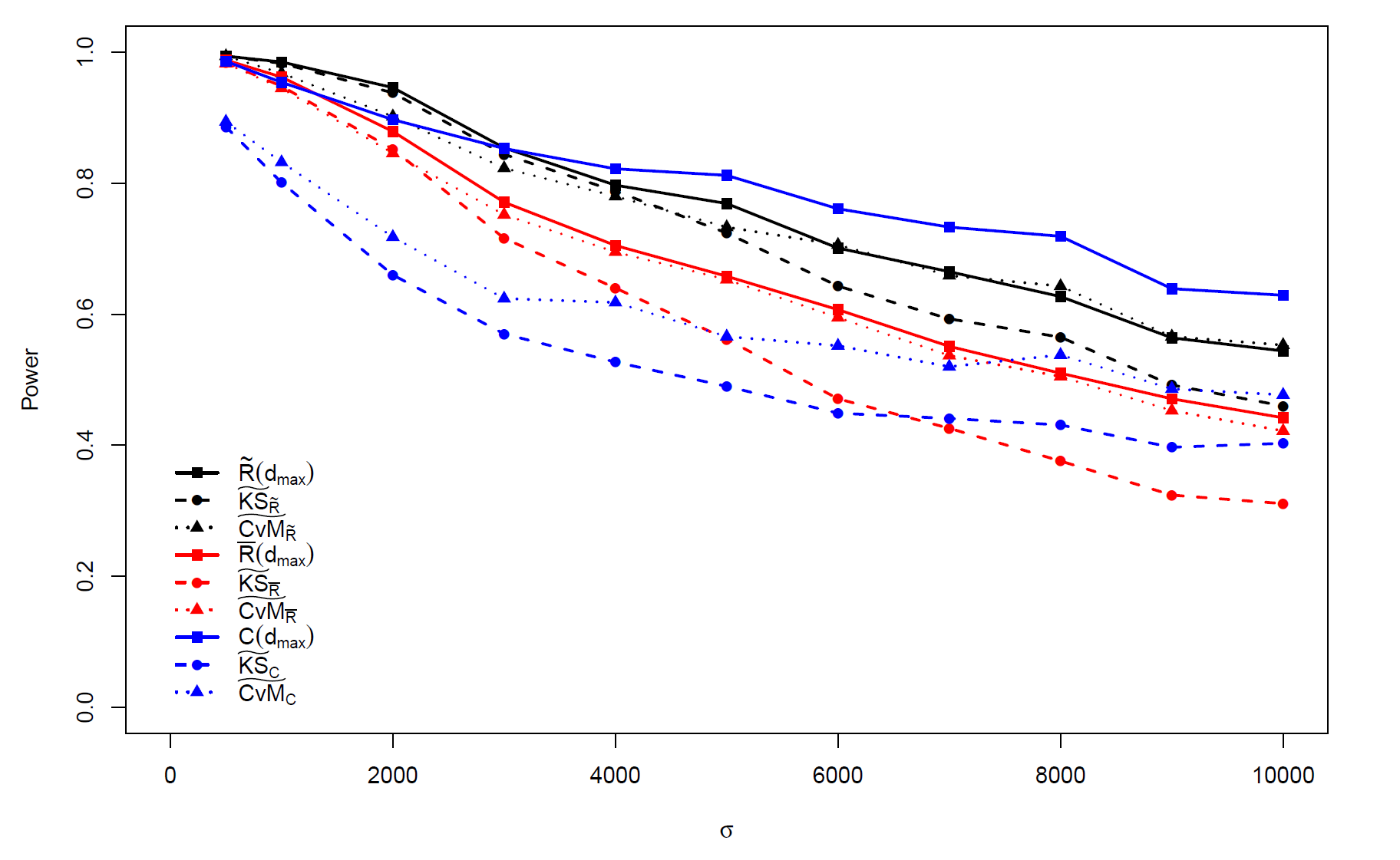

Supplement: S2 Fig — Only maximum powers of R¯(d), R˜(d), and C(d) over values of d considered are displayed; dmax refers to the value of d yielding the largest power. (TIF) [file pone.0204156.s002.tif]

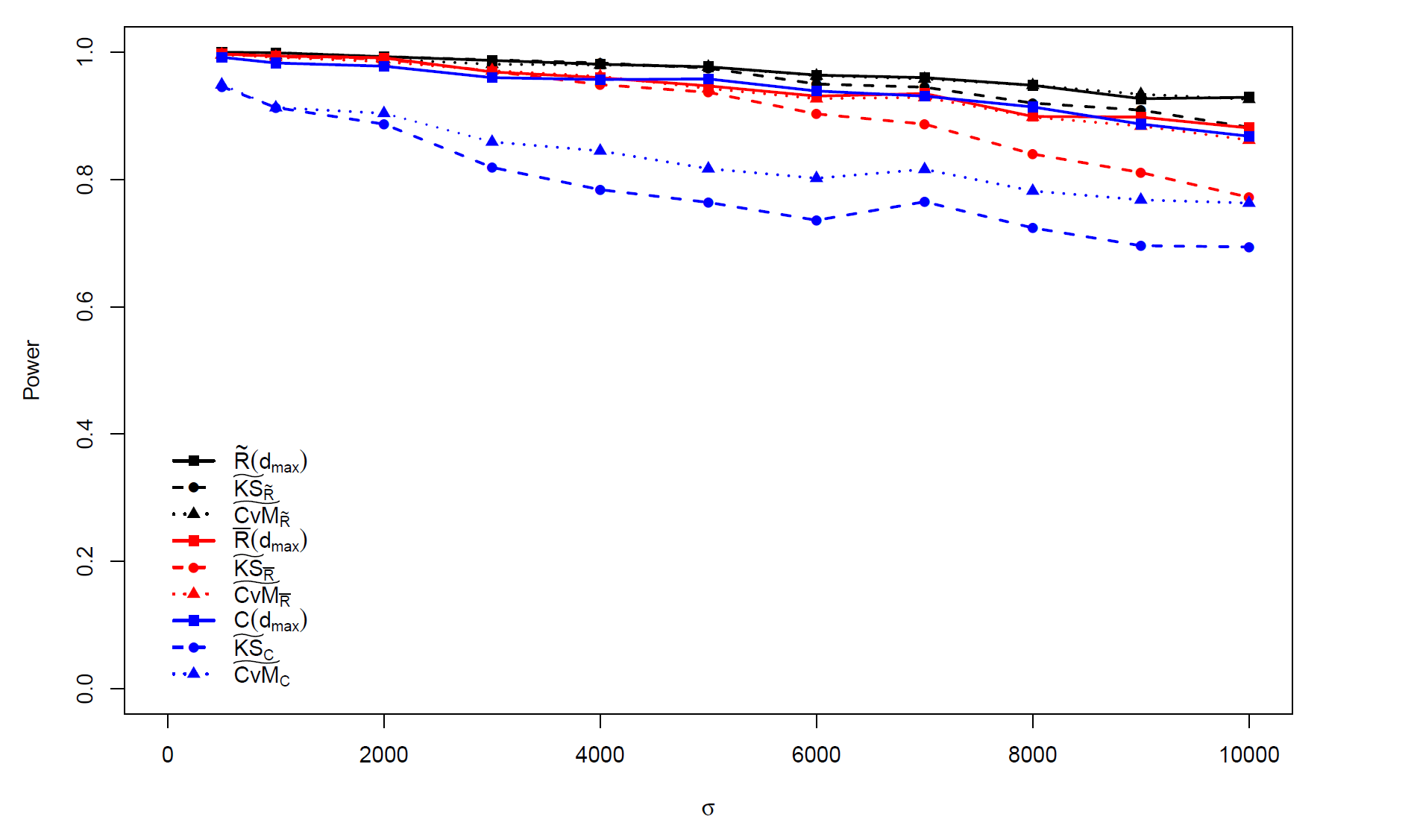

Supplement: S3 Fig — Only maximum powers of R¯(d), R˜(d), and C(d) over values of d considered are displayed; dmax refers to the value of d yielding the largest power. σ. (TIF) [file pone.0204156.s003.TIF]

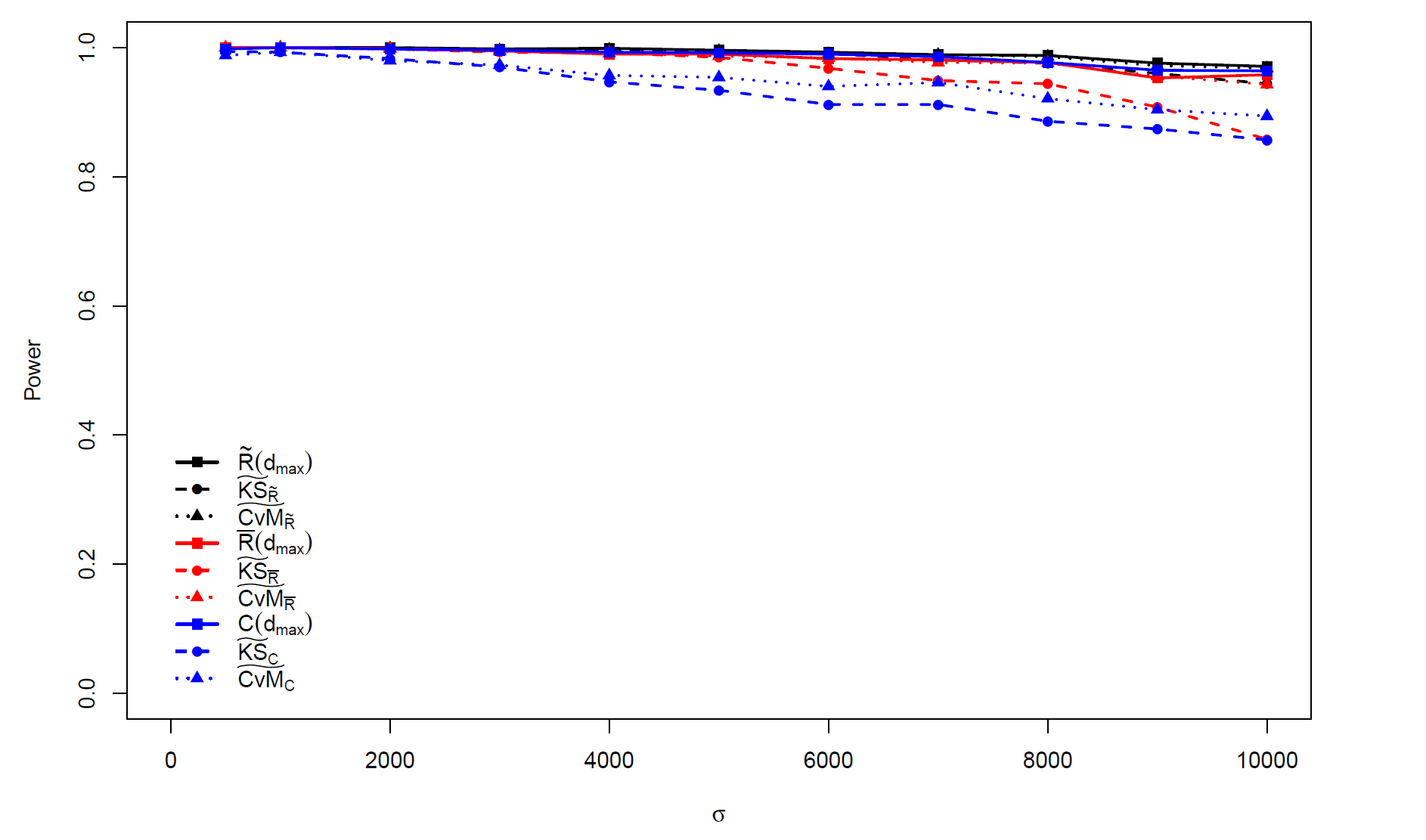

Supplement: S4 Fig — Only maximum powers of R¯(d), R˜(d), and C(d) over values of d considered are displayed; dmax refers to the value of d yielding the largest power. σ. (TIF) [file pone.0204156.s004.TIF]

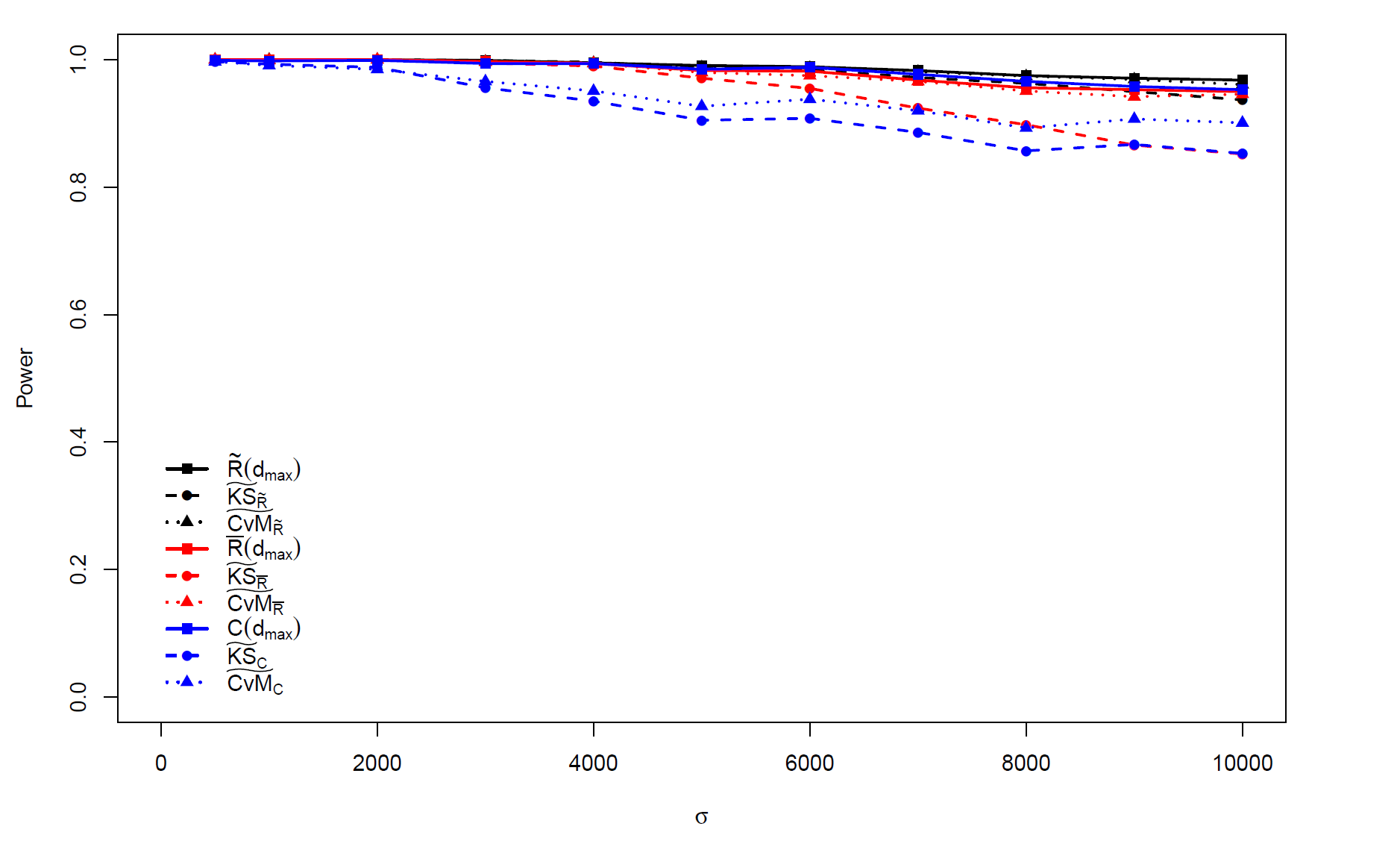

Supplement: S5 Fig — Only maximum powers of R¯(d), R˜(d), and C(d) over values of d considered are displayed; dmax refers to the value of d yielding the largest power. σ. (TIF) [file pone.0204156.s005.TIF]
