# Supplementary material for: Spatial statistical tools for genome-wide mutation cluster detection under a microarray probe sampling system
Source: PLoS One. 2018 Sep 25;13(9):e0204156. doi: 10.1371/journal.pone.0204156 (PMC6155535; doi:10.1371/journal.pone.0204156)
Supplement: S1 Table — Under each parameter setting, h is set as h = 3σ and μp is set to match with η = 50. For R¯(d), R˜(d), Dmin(n), Nmax(d) and C(d), only the maximum power across the values considered for d or n is shown. The significance level of the test is set as α = 0.05. (PDF) [file pone.0204156.s006.pdf]

Table S1: Power of the tests under alternative hypothesis (1) with $\mu_o = 375$ under various σ choices.

**Parameter settings**	**1**	**2**	**3**	**4**	**5**	**6**	**7**	**8**	**9**	**10**	**11**
μ_p	767	726	715	704	701	702	702	696	695	692	699
μ_o	375	375	375	375	375	375	375	375	375	375	375
σ	500	1000	2000	3000	4000	5000	6000	7000	8000	9000	10000
h	1500	3000	6000	9000	12000	15000	18000	21000	24000	27000	30000
**Test statistics**											
$\bar{R}(d)$ MAX	0.926	0.852	0.724	0.622	0.549	0.504	0.469	0.458	0.407	0.361	0.374
$\widetilde{KS}_{\bar{R}}$	0.919	0.827	0.707	0.599	0.514	0.441	0.417	0.399	0.338	0.256	0.273
$\widetilde{CvM}_{\bar{R}}$	0.888	0.793	0.695	0.611	0.544	0.497	0.486	0.458	0.403	0.341	0.361
$\tilde{R}(d)$ **MAX**	0.951	0.901	0.815	0.734	0.661	0.607	0.566	0.564	0.507	0.440	0.434
$\widetilde{KS}_{\tilde{R}}$	0.946	0.898	0.813	0.739	0.653	0.603	0.538	0.511	0.473	0.370	0.360
$\widetilde{CvM}_{\tilde{R}}$	0.926	0.852	0.751	0.702	0.624	0.590	0.550	0.535	0.503	0.429	0.433
$D_{min}(n)$ **MAX**	0.860	0.678	0.475	0.355	0.290	0.261	0.242	0.242	0.213	0.206	0.214
$\widetilde{KS}_{D_{min}}$	0.076	0.064	0.057	0.058	0.072	0.069	0.052	0.059	0.065	0.062	0.052
$\widetilde{CvM}_{D_{min}}$	0.081	0.060	0.057	0.063	0.073	0.073	0.057	0.057	0.069	0.055	0.049
$N_{max}(d)$ **MAX**	0.438	0.364	0.285	0.249	0.252	0.240	0.229	0.223	0.199	0.189	0.198
$\widetilde{KS}_{N_{max}}$	0.447	0.361	0.296	0.242	0.234	0.233	0.220	0.216	0.188	0.181	0.178
$\widetilde{CvM}_{N_{max}}$	0.431	0.342	0.279	0.230	0.223	0.222	0.205	0.202	0.177	0.165	0.162
$C(d)$ MAX	0.873	0.785	0.708	0.643	0.581	0.582	0.555	0.530	0.488	0.419	0.417
$\widetilde{KS}_C$	0.595	0.472	0.398	0.305	0.285	0.281	0.283	0.244	0.238	0.226	0.247
$\widetilde{CvM}_C$	0.656	0.529	0.460	0.378	0.369	0.362	0.359	0.324	0.294	0.281	0.313

Under each parameter setting, h is set as $h = 3\sigma$ and μ_p is set to match with $\eta = 50$. For $\bar{R}(d)$, $\tilde{R}(d)$, $D_{min}(n)$, $N_{max}(d)$ and $C(d)$, only the maximum power across the values considered for d or n is shown. The significance level of the test is set as $\alpha = 0.05$.
