# Supplementary material for: Spatial statistical tools for genome-wide mutation cluster detection under a microarray probe sampling system
Source: PLoS One. 2018 Sep 25;13(9):e0204156. doi: 10.1371/journal.pone.0204156 (PMC6155535; doi:10.1371/journal.pone.0204156)
Supplement: S2 Table — Under each parameter setting, h is set as h = 3σ and μp is set to match with η = 50. For R¯(d), R˜(d), Dmin(n), Nmax(d) and C(d), only the maximum power across the values considered for d or n is shown. The significance level of the test is set as α = 0.05. (PDF) [file pone.0204156.s007.pdf]

Table S2: Power of the tests under alternative hypothesis (2) with $\mu_o = 375$ under various σ choices.

**Parameter settings**	**1**	**2**	**3**	**4**	**5**	**6**	**7**	**8**	**9**	**10**	**11**
μ_p	104	156	233	284	326	361	387	409	432	450	465
μ_o	375	375	375	375	375	375	375	375	375	375	375
σ	500	1000	2000	3000	4000	5000	6000	7000	8000	9000	10000
h	1500	3000	6000	9000	12000	15000	18000	21000	24000	27000	30000
**Test statistics**											
$\bar{R}(d)$ MAX	0.993	0.968	0.910	0.792	0.722	0.665	0.603	0.575	0.530	0.507	0.473
$\widetilde{KS}_{\bar{R}}$	0.986	0.957	0.889	0.763	0.649	0.573	0.502	0.437	0.390	0.351	0.338
$\widetilde{CvM}_{\bar{R}}$	0.988	0.952	0.875	0.782	0.711	0.651	0.587	0.560	0.509	0.480	0.465
$\tilde{R}(d)$ **MAX**	0.996	0.987	0.952	0.878	0.824	0.755	0.707	0.685	0.661	0.617	0.577
$\widetilde{KS}_{\tilde{R}}$	0.996	0.987	0.945	0.865	0.804	0.737	0.685	0.640	0.582	0.531	0.475
$\widetilde{CvM}_{\tilde{R}}$	0.995	0.971	0.917	0.853	0.795	0.740	0.701	0.690	0.642	0.622	0.584
$D_{min}(n)$ **MAX**	0.987	0.878	0.693	0.529	0.440	0.419	0.361	0.365	0.342	0.364	0.357
$\widetilde{KS}_{D_{min}}$	0.109	0.091	0.078	0.083	0.077	0.088	0.079	0.073	0.066	0.072	0.060
$\widetilde{CvM}_{D_{min}}$	0.114	0.099	0.074	0.096	0.075	0.086	0.084	0.072	0.065	0.070	0.066
$N_{max}(d)$ **MAX**	0.700	0.618	0.498	0.447	0.423	0.405	0.346	0.347	0.332	0.348	0.337
$\widetilde{KS}_{N_{max}}$	0.712	0.617	0.493	0.439	0.429	0.414	0.335	0.342	0.344	0.341	0.328
$\widetilde{CvM}_{N_{max}}$	0.698	0.605	0.484	0.426	0.408	0.398	0.322	0.324	0.328	0.317	0.309
$C(d)$ MAX	0.987	0.960	0.919	0.864	0.830	0.784	0.774	0.761	0.725	0.672	0.643
$\widetilde{KS}_{C}$	0.908	0.823	0.703	0.606	0.540	0.492	0.440	0.437	0.426	0.426	0.417
$\widetilde{CvM}_{C}$	0.915	0.845	0.743	0.672	0.605	0.582	0.546	0.538	0.508	0.503	0.494

Under each parameter setting, h is set as $h = 3\sigma$ and μ_p is set to match with $\eta = 50$. For $\bar{R}(d)$, $\tilde{R}(d)$, $D_{min}(n)$, $N_{max}(d)$ and $C(d)$, only the maximum power across the values considered for d or n is shown. The significance level of the test is set as $\alpha = 0.05$.
