# Supplementary material for: Spatial statistical tools for genome-wide mutation cluster detection under a microarray probe sampling system
Source: PLoS One. 2018 Sep 25;13(9):e0204156. doi: 10.1371/journal.pone.0204156 (PMC6155535; doi:10.1371/journal.pone.0204156)
Supplement: S3 Table — Under each parameter setting, h is set as h = 3σ and μp is set to match with η = 50. For R¯(d), R˜(d), Dmin(n), Nmax(d) and C(d), only the maximum power across the values considered for d or n is shown. The significance level of the test is set as α = 0.05. (PDF) [file pone.0204156.s008.pdf]

Table S3: Power of the tests under alternative hypothesis (3) with $\mu_o = 375$ under various σ choices.

Parameter settings	1	2	3	4	5	6	7	8	9	10	11
μ_p	115	172	252	309	347	384	410	434	454	472	487
μ_o	375	375	375	375	375	375	375	375	375	375	375
σ	500	1000	2000	3000	4000	5000	6000	7000	8000	9000	10000
h	1500	3000	6000	9000	12000	15000	18000	21000	24000	27000	30000
**Test statistics**											
$\bar{R}(d)$ MAX	0.988	0.962	0.879	0.771	0.705	0.658	0.607	0.551	0.510	0.471	0.442
$\widetilde{KS}_{\bar{R}}$	0.984	0.948	0.852	0.716	0.640	0.562	0.471	0.426	0.376	0.324	0.311
$\widetilde{CvM}_{\bar{R}}$	0.983	0.945	0.846	0.752	0.695	0.653	0.595	0.537	0.505	0.453	0.422
$\tilde{R}(d)$ **MAX**	0.994	0.985	0.946	0.854	0.797	0.769	0.701	0.665	0.627	0.564	0.544
$\widetilde{KS}_{\tilde{R}}$	0.994	0.983	0.938	0.844	0.788	0.724	0.643	0.593	0.565	0.492	0.460
$\widetilde{CvM}_{\tilde{R}}$	0.994	0.968	0.902	0.823	0.780	0.733	0.706	0.659	0.643	0.566	0.553
$D_{min}(n)$ **MAX**	0.966	0.872	0.653	0.472	0.418	0.387	0.388	0.369	0.358	0.336	0.339
$\widetilde{KS}_{D_{min}}$	0.104	0.111	0.075	0.077	0.060	0.075	0.070	0.076	0.065	0.055	0.056
$\widetilde{CvM}_{D_{min}}$	0.116	0.108	0.070	0.083	0.063	0.084	0.078	0.080	0.071	0.058	0.059
$N_{max}(d)$ **MAX**	0.687	0.585	0.482	0.416	0.398	0.364	0.372	0.353	0.337	0.316	0.321
$\widetilde{KS}_{N_{max}}$	0.687	0.589	0.457	0.433	0.396	0.374	0.365	0.355	0.324	0.312	0.295
$\widetilde{CvM}_{N_{max}}$	0.679	0.578	0.450	0.410	0.380	0.362	0.352	0.341	0.313	0.292	0.278
$C(d)$ MAX	0.986	0.954	0.897	0.853	0.822	0.812	0.761	0.733	0.719	0.639	0.629
$\widetilde{KS}_C$	0.886	0.801	0.660	0.570	0.527	0.490	0.449	0.441	0.431	0.397	0.403
$\widetilde{CvM}_C$	0.894	0.832	0.718	0.624	0.618	0.566	0.552	0.520	0.538	0.486	0.477

Under each parameter setting, h is set as $h = 3\sigma$ and μ_p is set to match with $\eta = 50$. For $\bar{R}(d)$, $\tilde{R}(d)$, $D_{min}(n)$, $N_{max}(d)$ and $C(d)$, only the maximum power across the values considered for d or n is shown. The significance level of the test is set as $\alpha = 0.05$.
