# Supplementary material for: Spatial statistical tools for genome-wide mutation cluster detection under a microarray probe sampling system
Source: PLoS One. 2018 Sep 25;13(9):e0204156. doi: 10.1371/journal.pone.0204156 (PMC6155535; doi:10.1371/journal.pone.0204156)
Supplement: S4 Table — Under each parameter setting, h is set as h = 3σ and μp is set to match with η = 50. For R¯(d), R˜(d), Dmin(n), Nmax(d) and C(d), only the maximum power across the values considered for d or n is shown. The significance level of the test is set as α = 0.05. (PDF) [file pone.0204156.s009.pdf]

Table S4: Power of the tests under alternative hypothesis (1) with $\mu_o = 1125$ under various σ choices.

**Parameter settings**	**1**	**2**	**3**	**4**	**5**	**6**	**7**	**8**	**9**	**10**	**11**
μ_p	309	270	251	244	241	236	238	238	237	235	235
μ_o	1125	1125	1125	1125	1125	1125	1125	1125	1125	1125	1125
σ	500	1000	2000	3000	4000	5000	6000	7000	8000	9000	10000
h	1500	3000	6000	9000	12000	15000	18000	21000	24000	27000	30000
**Test statistics**											
$\bar{R}(d)$ MAX	0.997	0.994	0.991	0.969	0.960	0.947	0.931	0.935	0.899	0.898	0.881
$\widetilde{KS}_{\bar{R}}$	0.997	0.993	0.989	0.970	0.949	0.937	0.903	0.887	0.840	0.811	0.772
$\widetilde{CvM}_{\bar{R}}$	0.996	0.992	0.985	0.971	0.962	0.943	0.927	0.929	0.898	0.884	0.862
$\tilde{R}(d)$ MAX	1.000	0.999	0.993	0.987	0.981	0.977	0.964	0.960	0.948	0.927	0.929
$\widetilde{KS}_{\tilde{R}}$	0.999	0.999	0.993	0.988	0.983	0.975	0.950	0.945	0.920	0.909	0.882
$\widetilde{CvM}_{\tilde{R}}$	0.996	0.995	0.989	0.981	0.980	0.977	0.963	0.958	0.948	0.934	0.926
$D_{min}(n)$ MAX	0.986	0.925	0.803	0.667	0.631	0.607	0.601	0.624	0.542	0.557	0.582
$\widetilde{KS}_{D_{min}}$	0.141	0.139	0.108	0.102	0.080	0.099	0.099	0.081	0.079	0.078	0.073
$\widetilde{CvM}_{D_{min}}$	0.142	0.147	0.111	0.118	0.092	0.097	0.103	0.090	0.084	0.084	0.076
$N_{max}(d)$ MAX	0.827	0.762	0.704	0.652	0.623	0.598	0.579	0.614	0.526	0.540	0.565
$\widetilde{KS}_{N_{max}}$	0.830	0.758	0.712	0.643	0.639	0.589	0.583	0.610	0.529	0.530	0.548
$\widetilde{CvM}_{N_{max}}$	0.825	0.753	0.704	0.634	0.629	0.576	0.569	0.603	0.513	0.514	0.529
$C(d)$ MAX	0.992	0.983	0.978	0.960	0.957	0.958	0.939	0.931	0.914	0.887	0.868
$\widetilde{KS}_{C}$	0.946	0.913	0.887	0.819	0.784	0.764	0.736	0.765	0.724	0.696	0.694
$\widetilde{CvM}_{C}$	0.949	0.913	0.904	0.859	0.845	0.817	0.802	0.816	0.782	0.768	0.763

Under each parameter setting, h is set as $h = 3\sigma$ and μ_p is set to match with $\eta = 50$. For $\bar{R}(d)$, $\tilde{R}(d)$, $D_{min}(n)$, $N_{max}(d)$ and $C(d)$, only the maximum power across the values considered for d or n is shown. The significance level of the test is set as $\alpha = 0.05$.
