# Supplementary material for: Spatial statistical tools for genome-wide mutation cluster detection under a microarray probe sampling system
Source: PLoS One. 2018 Sep 25;13(9):e0204156. doi: 10.1371/journal.pone.0204156 (PMC6155535; doi:10.1371/journal.pone.0204156)
Supplement: S5 Table — Under each parameter setting, h is set as h = 3σ and μp is set to match with η = 50. For R¯(d), R˜(d), Dmin(n), Nmax(d) and C(d), only the maximum power across the values considered for d or n is shown. The significance level of the test is set as α = 0.05. (PDF) [file pone.0204156.s010.pdf]

Table S5: Power of the tests under alternative hypothesis (2) with $\mu_o = 1125$ under various σ choices.

Parameter settings	1	2	3	4	5	6	7	8	9	10	11
μ_p	44	60	83	99	112	123	131	139	147	151	156
μ_o	1125	1125	1125	1125	1125	1125	1125	1125	1125	1125	1125
σ	500	1000	2000	3000	4000	5000	6000	7000	8000	9000	10000
h	1500	3000	6000	9000	12000	15000	18000	21000	24000	27000	30000
**Test statistics**											
$\bar{R}(d)$ MAX	1.000	1.000	0.998	0.995	0.990	0.990	0.983	0.981	0.977	0.953	0.958
$\widetilde{KS}_{\bar{R}}$	1.000	1.000	0.998	0.993	0.992	0.985	0.968	0.949	0.944	0.908	0.858
$\widetilde{CvM}_{\bar{R}}$	1.000	1.000	0.998	0.994	0.991	0.987	0.984	0.977	0.976	0.956	0.943
$\tilde{R}(d)$ MAX	1.000	1.000	1.000	0.998	0.999	0.996	0.993	0.989	0.988	0.976	0.971
$\widetilde{KS}_{\tilde{R}}$	1.000	1.000	1.000	0.997	0.997	0.995	0.991	0.981	0.976	0.960	0.944
$\widetilde{CvM}_{\tilde{R}}$	1.000	1.000	0.999	0.996	0.997	0.995	0.990	0.989	0.986	0.972	0.968
$D_{min}(n)$ MAX	0.998	0.992	0.937	0.877	0.819	0.814	0.783	0.773	0.755	0.718	0.725
$\widetilde{KS}_{D_{min}}$	0.269	0.268	0.187	0.171	0.159	0.147	0.129	0.138	0.118	0.158	0.109
$\widetilde{CvM}_{D_{min}}$	0.279	0.292	0.198	0.177	0.167	0.146	0.142	0.152	0.134	0.155	0.109
$N_{max}(d)$ MAX	0.976	0.952	0.911	0.872	0.810	0.805	0.775	0.770	0.750	0.715	0.721
$\widetilde{KS}_{N_{max}}$	0.976	0.951	0.912	0.880	0.815	0.809	0.780	0.768	0.742	0.702	0.708
$\widetilde{CvM}_{N_{max}}$	0.976	0.950	0.911	0.874	0.806	0.806	0.772	0.761	0.737	0.694	0.692
$C(d)$ MAX	0.998	1.000	0.998	0.996	0.993	0.992	0.990	0.986	0.977	0.965	0.964
$\widetilde{KS}_C$	0.994	0.993	0.983	0.970	0.947	0.934	0.912	0.912	0.886	0.874	0.857
$\widetilde{CvM}_C$	0.988	0.993	0.980	0.973	0.957	0.954	0.940	0.946	0.921	0.904	0.894

Under each parameter setting, h is set as $h = 3\sigma$ and μ_p is set to match with $\eta = 50$. For $\bar{R}(d)$, $\tilde{R}(d)$, $D_{min}(n)$, $N_{max}(d)$ and $C(d)$, only the maximum power across the values considered for d or n is shown. The significance level of the test is set as $\alpha = 0.05$.
