# Supplementary material for: Spatial statistical tools for genome-wide mutation cluster detection under a microarray probe sampling system
Source: PLoS One. 2018 Sep 25;13(9):e0204156. doi: 10.1371/journal.pone.0204156 (PMC6155535; doi:10.1371/journal.pone.0204156)
Supplement: S6 Table — Under each parameter setting, h is set as h = 3σ and μp is set to match with η = 50. For R¯(d), R˜(d), Dmin(n), Nmax(d) and C(d), only the maximum power across the values considered for d or n is shown. The significance level of the test is set as α = 0.05. (PDF) [file pone.0204156.s011.pdf]

**Table S6: Power of the tests under alternative hypothesis (3) with $\mu_o = 1125$ under various $\sigma$ choices.**

| Parameter settings | 1 | 2 | 3 | 4 | 5 | 6 | 7 | 8 | 9 | 10 | 11 |
|---|---|---|---|---|---|---|---|---|---|---|---|
| $\mu_p$ | 48 | 64 | 90 | 106 | 121 | 130 | 141 | 149 | 153 | 159 | 163 |
| $\mu_o$ | 1125 | 1125 | 1125 | 1125 | 1125 | 1125 | 1125 | 1125 | 1125 | 1125 | 1125 |
| $\sigma$ | 500 | 1000 | 2000 | 3000 | 4000 | 5000 | 6000 | 7000 | 8000 | 9000 | 10000 |
| $h$ | 1500 | 3000 | 6000 | 9000 | 12000 | 15000 | 18000 | 21000 | 24000 | 27000 | 30000 |
| **Test statistics** | | | | | | | | | | | |
| $\bar{R}(d)$ MAX | 1.000 | 1.000 | 1.000 | 0.997 | 0.994 | 0.983 | 0.982 | 0.968 | 0.956 | 0.953 | 0.950 |
| $\widetilde{KS}_{\bar{R}}$ | 1.000 | 0.999 | 1.000 | 0.995 | 0.990 | 0.971 | 0.955 | 0.924 | 0.898 | 0.866 | 0.852 |
| $\widetilde{CvM}_{\bar{R}}$ | 1.000 | 0.999 | 1.000 | 0.996 | 0.994 | 0.980 | 0.975 | 0.966 | 0.951 | 0.942 | 0.946 |
| $\tilde{R}(d)$ **MAX** | 1.000 | 1.000 | 1.000 | 0.999 | 0.995 | 0.991 | 0.989 | 0.983 | 0.975 | 0.971 | 0.968 |
| $\widetilde{KS}_{\tilde{R}}$ | 1.000 | 1.000 | 1.000 | 0.999 | 0.996 | 0.988 | 0.985 | 0.972 | 0.963 | 0.950 | 0.937 |
| $\widetilde{CvM}_{\tilde{R}}$ | 1.000 | 1.000 | 1.000 | 0.997 | 0.995 | 0.988 | 0.987 | 0.981 | 0.974 | 0.969 | 0.961 |
| $D_{min}(n)$ **MAX** | 1.000 | 0.987 | 0.918 | 0.872 | 0.830 | 0.781 | 0.767 | 0.760 | 0.739 | 0.721 | 0.694 |
| $\widetilde{KS}_{D_{min}}$ | 0.276 | 0.234 | 0.180 | 0.187 | 0.154 | 0.144 | 0.117 | 0.118 | 0.115 | 0.135 | 0.102 |
| $\widetilde{CvM}_{D_{min}}$ | 0.291 | 0.242 | 0.192 | 0.201 | 0.170 | 0.156 | 0.133 | 0.125 | 0.134 | 0.130 | 0.107 |
| $N_{max}(d)$ **MAX** | 0.980 | 0.942 | 0.890 | 0.868 | 0.824 | 0.773 | 0.759 | 0.751 | 0.729 | 0.711 | 0.680 |
| $\widetilde{KS}_{N_{max}}$ | 0.980 | 0.944 | 0.892 | 0.876 | 0.836 | 0.778 | 0.769 | 0.751 | 0.714 | 0.698 | 0.669 |
| $\widetilde{CvM}_{N_{max}}$ | 0.980 | 0.940 | 0.889 | 0.869 | 0.832 | 0.773 | 0.761 | 0.742 | 0.711 | 0.691 | 0.655 |
| $C(d)$ MAX | 0.999 | 0.998 | 0.999 | 0.994 | 0.994 | 0.985 | 0.988 | 0.977 | 0.966 | 0.958 | 0.953 |
| $\widetilde{KS}_C$ | 0.997 | 0.993 | 0.988 | 0.956 | 0.935 | 0.905 | 0.908 | 0.886 | 0.857 | 0.867 | 0.853 |
| $\widetilde{CvM}_C$ | 0.997 | 0.991 | 0.985 | 0.966 | 0.951 | 0.927 | 0.938 | 0.920 | 0.893 | 0.907 | 0.901 |

Under each parameter setting, $h$ is set as $h = 3\sigma$ and $\mu_p$ is set to match with $\eta = 50$. For $\bar{R}(d)$, $\tilde{R}(d)$, $D_{min}(n)$, $N_{max}(d)$ and $C(d)$, only the maximum power across the values considered for $d$ or $n$ is shown. The significance level of the test is set as $\alpha = 0.05$.
