# Supplementary material for: Spatial statistical tools for genome-wide mutation cluster detection under a microarray probe sampling system
Source: PLoS One. 2018 Sep 25;13(9):e0204156. doi: 10.1371/journal.pone.0204156 (PMC6155535; doi:10.1371/journal.pone.0204156)
Supplement: S9 Table — Optimal argument settings of d or n for Neyman-Scott (NS) process under alternative hypothesis (3) with μo = 375 under various σ choices. Under each parameter setting, h is set as h = 3σ and μp is set to match with η = 50. (PDF) [file pone.0204156.s014.pdf]

Table S9: Optimal argument settings under alternative hypothesis (3) with $\mu_o = 375$.

**Parameter settings**	**1**	**2**	**3**	**4**	**5**	**6**	**7**	**8**	**9**	**10**	**11**
$\boldsymbol{\mu_p}$	115	172	252	309	347	384	410	434	454	472	487
$\boldsymbol{\mu_o}$	375	375	375	375	375	375	375	375	375	375	375
$\boldsymbol{\sigma}$	500	1000	2000	3000	4000	5000	6000	7000	8000	9000	10000
$\boldsymbol{h}$	1500	3000	6000	9000	12000	15000	18000	21000	24000	27000	30000
**Test statistics**											
$\boldsymbol{\bar{R}(d)}$	5000	5000	5000	10000	10000	15000	15000	20000	25000	25000	30000
$\boldsymbol{\tilde{R}(d)}$	5000	5000	5000	10000	10000	10000	15000	15000	25000	20000	25000
$\boldsymbol{D_{min}(n)}$	2	2	2	2	3	3	3	3	3	3	3
$\boldsymbol{N_{max}(d)}$	30000	30000	30000	30000	30000	30000	30000	30000	30000	30000	30000
$\boldsymbol{C(d)}$	15000	15000	15000	15000	15000	15000	15000	15000	15000	15000	15000

Optimal argument settings of d or n for Neyman-Scott (NS) process under alternative hypothesis (3) with $\mu_o = 375$ under various σ choices. Under each parameter setting, h is set as $h = 3\sigma$ and μ_p is set to match with $\eta = 50$.
