# Supplementary material for: Spatial statistical tools for genome-wide mutation cluster detection under a microarray probe sampling system
Source: PLoS One. 2018 Sep 25;13(9):e0204156. doi: 10.1371/journal.pone.0204156 (PMC6155535; doi:10.1371/journal.pone.0204156)
Supplement: S12 Table — Optimal argument settings of d or n for Neyman-Scott (NS) process under alternative hypothesis (3) with μo = 1125 under various σ choices. Under each parameter setting, h is set as h = 3σ and μp is set to match with η = 50. (PDF) [file pone.0204156.s017.pdf]

Table S12: Optimal argument settings under alternative hypothesis (3) with $\mu_o = 1125$.

**Parameter settings**	**1**	**2**	**3**	**4**	**5**	**6**	**7**	**8**	**9**	**10**	**11**
μ_p	48	64	90	106	121	130	141	149	153	159	163
μ_o	1125	1125	1125	1125	1125	1125	1125	1125	1125	1125	1125
σ	500	1000	2000	3000	4000	5000	6000	7000	8000	9000	10000
h	1500	3000	6000	9000	12000	15000	18000	21000	24000	27000	30000
**Test statistics**											
$\bar{R}(d)$	5000	50000	5000	5000	15000	15000	20000	25000	25000	20000	25000
$\tilde{R}(d)$	5000	5000	5000	5000	15000	10000	15000	20000	15000	25000	25000
$D_{min}(n)$	2	2	2	3	3	3	3	3	3	3	3
$N_{max}(d)$	30000	30000	30000	30000	30000	30000	30000	30000	30000	30000	30000
$C(d)$	5000	5000	10000	10000	15000	15000	15000	15000	15000	25000	15000

Optimal argument settings of d or n for Neyman-Scott (NS) process under alternative hypothesis (3) with $\mu_o = 1125$ under various σ choices. Under each parameter setting, h is set as $h = 3\sigma$ and μ_p is set to match with $\eta = 50$.
